# Towards the complete small RNome of *Acinetobacter baumannii*

**DOI:** 10.1099/mgen.0.000045

**Published:** 2016-03-02

**Authors:** Andy Weiss, William H. Broach, Mackenzie C. Lee, Lindsey N. Shaw

**Affiliations:** Cell Biology, Microbiology & Molecular Biology, University of South Florida, 4202 East Fowler Avenue, Tampa, FL 33620, USA

**Keywords:** *Acinetobacter baumannii*, C4 antisense RNA, RNA-seq, sRNA, sRNA annotation, sRNA modularity, small regulatory RNA

## Abstract

In recent years, the Gram-negative bacterium *Acinetobacter baumannii* has garnered considerable attention for its unprecedented capacity to rapidly develop resistance to antibacterial therapeutics. This is coupled with the seemingly epidemic emergence of new hyper-virulent strains. Although strain-specific differences for *A. baumannii* isolates have been well described, these studies have primarily focused on proteinaceous factors. At present, only limited publications have investigated the presence and role of small regulatory RNA (sRNA) transcripts. Herein, we perform such an analysis, describing the RNA-seq-based identification of 78 *A. baumannii* sRNAs in the AB5075 background. Together with six previously identified elements, we include each of these in a new genome annotation file, which will serve as a tool to investigate regulatory events in this organism. Our work reveals that the sRNAs display high expression, accounting for >50 % of the 20 most strongly expressed genes. Through conservation analysis we identified six classes of similar sRNAs, with one found to be particularly abundant and homologous to regulatory, C4 antisense RNAs found in bacteriophages. These elements appear to be processed from larger transcripts in an analogous manner to the phage C4 molecule and are putatively controlled by two further sRNAs that are strongly antisense to them. Collectively, this study offers a detailed view of the sRNA content of *A. baumannii*, exposing sequence and structural conservation amongst these elements, and provides novel insight into the potential evolution, and role, of these understudied regulatory molecules.

## Data Summary

1. The RNA-seq results have been deposited to the NCBI Gene Expression Omnibus; GEO submission GSE75708: http://www.ncbi.nlm.nih.gov/geo/query/acc.cgi?token = erytkcwadrsfdqv&acc = GSE75708
2. The updated GenBank file containing novel sRNA annotations (annotated as ‘misc. RNA’) has been deposited to Figshare: http://dx.doi.org/10.6084/m9.figshare.1592959

## Impact Statement

*Acinetobacter baumannii* is an emerging pathogen with tremendous genetic flexibility and a strong propensity towards the development of multidrug resistance. In particular, recent clinical isolates such as AB5075 display the potential for fatal infections and large-scale outbreaks. Although extensive work has been performed to unravel the mechanisms of multidrug resistance and enhanced virulence within these strains, the ever-growing class of small regulatory RNAs (sRNAs) has so far been largely overlooked. sRNAs have been associated with the regulation of virulence- and lifestyle-associated processes in a large number of bacteria, and may serve as important clinical targets in our efforts to combat increasingly prevalent multidrug-resistant strains. Thus, our thorough characterization of the small RNome in this study has the potential to inform further investigation into the physiology and virulence of this organism. We also demonstrate remarkable conservation of sRNA secondary structure and motifs, and characterize a large class of phage-derived transcripts. These findings lay a foundation for future studies into sRNA biology in *A. baumannii* specifically and for the bacterial kingdom at large.

## Introduction

The opportunistic human pathogen *Acinetobacter baumannii* is characterized by an inherent genomic flexibility that has resulted in resistance to all current antibiotic treatments. Even amongst the ESKAPE pathogens (Rice, 2008), whose innate propensity toward antibiotic resistance currently threatens the viability of modern treatment options, *A. baumannii* is often one of the first species to develop resistance to new drugs (Pendleton *et al.*, 2013; Queenan & Bush, 2007). Indeed, *A. baumannii* strains with resistance to the most commonly used antimicrobials are frequently identified, including those lacking sensitivity to carbapenems, colistin, polymyxin B and tigecycline (Gales *et al.*, 2006; Li *et al.*, 2006; Ko *et al.*, 2007; Lesho *et al.*, 2013; Navon-Venezia *et al.*, 2007; Peleg *et al.*, 2008; Ruzin *et al.*, 2007; Vila *et al.*, 2007). The implications of such multidrug-resistant (MDR) strains in clinical settings is severe, where they are associated with ventilator-associated pneumonia, bloodstream infections, skin/soft tissue infections (e.g. in traumatic wound and burn victims), urinary tract infections and post-neurosurgical meningitis (Peleg *et al.*, 2008). Notably, a high incidence of infection has also been demonstrated for soldiers returning from duty in Afghanistan and Iraq, often in the form of osteomyelitis following bone fractures (Davis *et al.*, 2005; Hujer *et al.*, 2006; Yun *et al.*, 2008).

Although considered to pose a lower risk to immunocompetent patients due to its higher tendency toward colonization over infection, *A. baumannii* possesses a range of virulence genes that may be important in its clinical pathogenicity. However, most such factors found in the genomes of clinical isolates thus far are also present in the genome of the largely avirulent ATCC 17978 strain, isolated 50 years ago. This suggests that the recent surge in *A. baumannii* epidemics cannot solely be attributed to an expanded pathogenic repertoire (Imperi *et al.*, 2011). The pathogenicity of *A. baumannii* instead likely results from two factors: (1) its ability to survive in hospital environments for extended periods of time (Jawad *et al.*, 1998; Kramer *et al.*, 2006; Wisplinghoff *et al.*, 2007) and (2) an unusually high disposition towards assimilating foreign genetic elements into its accessory genome, which then function as regulatory, virulence and resistance factors, enhancing its pathogenic nature. Such exogenously acquired genetic units are, at least in part, responsible for the strain-specific differences seen with various *A. baumannii* isolates, complicating the selection of strains for an accurate model of pathogenesis. The highly virulent isolate AB5075 has recently been proposed as a model strain for pathogenic MDR *A. baumannii* (Jacobs *et al.*, 2014). Although many studies in the past have used classical ATCC laboratory strains (19606 and 17978), these were isolated decades ago, before the modern antibiotic resistance crisis. Thus, AB5075, first isolated in 2008 at a US military hospital, provides a more contemporary strain of choice for study. Evaluation of this isolate has shown that it accurately represents *A. baumannii* infections in multiple disease models, including a mouse pulmonary model, a *Galleria mellonella* model and a rat osteomyelitis model (Jacobs *et al.*, 2014).

Although strain-specific differences for various *A. baumannii* laboratory and clinical isolates have been well described, these studies have focused primarily on proteinaceous factors. At present, only limited publications have investigated the presence and role of small regulatory RNA (sRNA) transcripts. To the best of our knowledge, only one study to date has performed a systematic search to identify these molecules (Sharma *et al.*, 2014). Using bioinformatics approaches, 31 small transcripts were predicted, with three confirmed by experimental methods (Sharma *et al.*, 2014). Beyond this, the sRNA Aar has been described in the closely related bacterium *Acinetobacter baylyi*, with a homologous region in *A. baumannii* also identified (Schilling *et al.*, 2010). As sRNAs function as global regulators of bacterial physiology (Waters & Storz, 2009) and can play pivotal roles in the global regulation of virulence factors (Gripenland *et al.*, 2010; Romby *et al.*, 2006), developing a more comprehensive understanding of the *A. baumannii* sRNA content would be of considerable utility. By identifying and annotating sRNAs in multiple *A. baumannii* strains, we can begin to elucidate their roles and provide insight into strain-specific differences, particularly in the context of pathogenesis.

Herein, we perform such an analysis, describing the RNA-seq-based identification of 78 *A. baumannii* sRNA transcripts in the AB5075 background. Together with six previously known RNAs, we include each of them in a new genome annotation file, which will serve as an invaluable tool to investigate regulatory events in this organism. Our work reveals that these newly annotated genes display remarkably high expression, accounting for >50 % of the 20 most strongly expressed genes. Furthermore, we investigated the conservation of these sRNAs across *A. baumannii* strains, as well as one other member of the genus *Acinetobacter*. Through these analyses we identified several sRNAs that are present in large copy numbers within the AB5075 genome itself. These similar sRNAs are grouped into six classes, with one found to be particularly abundant and homologous to regulatory, C4 antisense RNAs in bacteriophages. Additionally, two sRNAs were found that are antisense to these phage-derived transcripts, giving them the potential to exert broad regulatory influence, thereby adding to the complexity of regulatory networks in *A. baumannii*. Collectively, this study offers a detailed view of the sRNA content of *A. baumannii*, exposing sequence and structural conservation amongst these elements, and provides insight into the potential evolution, and role, of these understudied regulatory molecules.

## Methods

### RNA-seq

RNA-seq experiments were performed as described by us previously for *Staphylococcus aureus* (Carroll *et al.*, 2016; Weiss *et al.*, 2014). Briefly, *A. baumannii* AB5075 was grown overnight at 37 °C in LB and diluted 1 : 100 into 100 ml fresh LB. Cultures were then grown into exponential phase and subsequently used to seed new cultures to OD_600_ 0.05. After growth for 3 h at 37 °C cultures were pelleted and the supernatant removed. Cells were stored at − 80 °C, prior to RNA isolation using an RNeasy kit (Qiagen). DNA was removed from samples using a TURBO DNA-free kit (Ambion). In order to confirm integrity of the RNA, sample quality was determined using an Agilent 2100 Bioanalyzer system and a corresponding RNA 6000 Nano kit (Agilent). Three biological replicates from this procedure were pooled and rRNA removed by sequential treatment with a Ribo-Zero bacterial rRNA Removal kit (Epicentre) and a MICROBExpress Bacterial mRNA enrichment kit (Agilent). The complete removal of rRNA was confirmed via analysis with a RNA 6000 Nano kit (Agilent). Enriched samples were then used as input material for RNA-seq using an Ion Torrent Personal Genome Machine. Briefly, strand-specific cDNA libraries were constructed using a Total RNA-seq kit v2 and its corresponding whole-transcriptome protocol (Ion Torrent). The quality and concentration of libraries was assessed with an Agilent Bioanalyzer system in combination with a High sensitivity DNA kit (Agilent). Adaptor-bound fragments were then attached to Ion Sphere Particles, amplified, and enriched using an Ion Personal Genome Machine Template OT2 200 kit (Ion Torrent) and an Ion OneTouch 2 system (Ion Torrent). Template-positive Ion Sphere Particles were loaded onto an Ion 318 v2 chip (Ion Torrent) and used for sequencing reaction with an Ion PGM Sequencing 200 kit v2 (Ion Torrent). After completion of each run, data were recovered and used for subsequent analysis. The data derived from this study was deposited into NCBI Gene Expression Omnibus.

### Bioinformatics, sRNA identification and genome annotation

The majority of the bioinformatic analyses, including RNA-seq analysis, sRNA secondary structure prediction and blast searches, were performed using the CLC Genomics Workbench software (CLC bio; Qiagen). Specifically, sequencing data in the fastq format were imported into the CLC Genomics Workbench with reads aligned to the AB5075 reference genome (GenBank accession number NZ_CP008706.1) using default settings for the ‘Map Reads to Reference’ tool. In order to identify unannotated (sRNA) genes the genome file was explored manually for reads that indicated transcriptional activity distinct from existing annotations. If ≥ 20 reads aligned to a position in the genome that either (1) did not contain any annotation (e.g. intergenic regions) or (2) where aligned reads were overlapping, or in antisense direction to, an annotated gene, then a new annotation was included in the file. Using this method, only reads that showed distinct and clearly independent expression patterns, e.g. intergenic reads that did not extend into adjacent annotations or reads that aligned to the opposite strand of a pre-existing annotation, were considered. We employed a conservative approach to the boundaries of each annotation (5′ and 3′ ends), which were defined by the longest reads for each end of a transcript. Using these guidelines, a revised AB5075 annotation file containing sRNA annotations was created and deposited to Figshare. In order to investigate new annotations for encoded proteins or peptides the CLC Genomics Workbench ORF Finder was used. Here, the most common start codons (ATG, GTG and TTG) were considered and an ORF size of ≥ 30 aa was used. Expression values for all genes, as well as sRNAs, were calculated as RPKM (reads per kilobase material per million reads) using the standard settings for ‘RNA-seq analysis’ in the CLC software. To investigate structural properties of newly described transcripts, the Predict secondary structure function of CLC Genomics Workbench was used with default settings. Additional secondary structure predictions were performed using the RNAfold web server (Hofacker, 2003) and mfold algorithm (Zuker, 2003). The conservation of sequences for newly annotated sRNAs was investigated using nucleotide blast searches against the different *Acinetobacter* genomes. All genome annotation files were retrieved from GenBank [accession numbers: NC_009085 (*A. baumannii* ATCC 17978), NC_011586 (*A. baumannii* AB0057), NC_010410 (*A. baumannii* AYE), NC_011595 (*A. baumannii* AB307-0294), and NC_010611 (*A. baylyi* ADP1)]. Sequences were considered similar if an *E*-value of ≤ 1 × 10^− 10^ ( ≥ 10 for negative log-transformed *E*-values) was returned. To evaluate the presence of similar sRNAs and/or specific sRNA motifs within the AB5075 genome, a ‘self-blast’ of each sRNA against its own genome was performed. Stretches of >30 nt were subject to further analysis and characterization for sequence and structural conservation.

### Northern blots

The identification of RNA transcripts was achieved via Northern blot as described by Caswell *et al.* (2012). Growth of bacterial cultures as well as RNA isolation and DNA depletion were performed as described for RNA-seq experiments. Isolated RNA (15 μg) was separated via gel electrophoresis on a 10 % polyacrylamide gel containing 7 M urea and 1 ×  TBE. After transfer of the separated samples to an Amersham Hybond-N+ membrane (GE Healthcare) by electroblotting, membranes were exposed to UV radiation in order to cross-link samples. Next, membranes were pre-hybridized for 1 h at 43 °C in ULTRAhyb-Oligo buffer (Ambion) and incubated (16 h, 43 °C) with [γ-^32^]ATP end-labelled oligonucleotides specific for each target RNA sequence (5′ → 3′ direction: ABUWs030, CACAGAGTACAATTGCGAAGCCACCAGC; ABUWs042, ACGTGTTTTTAGTGCGCTTAGGTAGA; ABUWs062, AGGATTCCTATTTGGTCTTGCTTCTG; ABUWs074, ATCGCTTCCGTTCTAACATCTTATC; ABUWs076, TTGGCGCTGTAGTTGTTGTTTATAG; ABUWs077, AGTTTCCTCTTTATTCACCTACATC). The labelling of oligonucleotides was performed using T4 polynucleotide kinase (Thermo Scientific). After overnight incubation, each membrane was washed with decreasing (2 × , 1 ×  and 0.5 × ) concentrations of SSC buffer (300 mM sodium chloride, 30 mM sodium citrate) for 30 min at 43 °C. Lastly, membranes were exposed to X-ray film for detection of sRNA transcripts.

## Results

### Exploring the sRNA content of *A. baumannii* AB5075

In order to discern the sRNA content of *A. baumannii* (strain AB5075) we performed RNA-seq on exponentially growing cultures and aligned reads to the existing genome annotation file. As the publicly available genome for strain AB5075 does not include any sRNA annotations, we identified a large number of reads aligning to areas of the genome that did not contain annotations. These were primarily found in intergenic regions, where we observed distinct expression patterns from adjacent genes. Additionally, we found a small number of sRNA genes overlapping or antisense to already existing annotations that again had visible differences in expression or read directionality compared with extant genes. Using this methodology, we were able to identify 78 novel transcripts under the conditions tested (Table 1). Two additional, previously documented but currently unannotated *Acinetobacter* sRNAs, Aar (Schilling *et al.*, 2010) and AbsR28 (Sharma *et al.*, 2014), as well as four highly conserved RNA species [the signal recognition particle (SRP) RNA, tmRNA, 6S RNA and RNase P RNA] were also identified using these methods. Thus, a total of 84 new sRNA annotations were introduced into the AB5075 genome file, increasing the number of genes by ∼2 %. The newly identified sRNAs were named Art1–Art78 (for *Acinetobacter* s*R*NA *T*ranscript). To further establish a nomenclature system that can be continuously extended and eliminates the problem of inconsistent naming for newly identified transcripts, we followed the approach used for protein-coding genes, which are named consecutively (ABUW_0001–ABUW_3906); thus, sRNA genes were termed ABUWs001–ABUWs084 (Table 1; the genomic context, including up- and downstream genes, is provided in Table S1, available in the online Supplementary Material).

**Table 1.** Annotated sRNA designations in AB5075

AB5075 designation	Gene name	Strand	Position	Size (nt)
ABUWs001	*art1*	>	92 978–93 245	268
ABUWs002	*art2*	<	154 776–154 945	170
ABUWs003	*art3*	>	175 358–175 585	228
ABUWs004	*art4*	<	233 515–233 635	121
ABUWs005	*art5*	>	236 876–237 005	130
ABUWs006	*art6*	>	244 194–244 272	79
ABUWs007	*art7*	>	407 081–407 297	217
ABUWs008	*art8*	>	449 632–449 749	118
ABUWs009	*art9*	>	549 559–549 856	298
ABUWs010	*art10*	<	555 198–555 404	207
ABUWs011	*art11*	<	629 088–629 256	169
ABUWs012	*absR28*	<	656 809–657 011	203
ABUWs013	*art12*	<	748 683–748 744	62
ABUWs014	*art13*	>	755 957–756 113	157
ABUWs015	*art14*	<	777 765–777 846	82
ABUWs016	*aar*	<	861 839–862 019	181
ABUWs017	*art15*	>	892 265–892 474	210
ABUWs018	*art16*	>	945 849–946 067	219
ABUWs019	*art17*	>	1 189 857–1 190 052	196
ABUWs020	*art18*	>	1 297 067–1 297 202	136
ABUWs021	*art19*	<	1 298 798–1 298 859	62
ABUWs022	*art20*	>	1 305 364–1 305 478	115
ABUWs023	*art21*	>	1 323 085–1 323 260	176
ABUWs024	*art22*	<	1 340 109–1 340 263	155
ABUWs025	*art23*	<	1 404 716–1 404 796	81
ABUWs026	*art24*	>	1 484 427–1 484 605	179
ABUWs027	*art25*	>	1 500 621–1 500 850	230
ABUWs028	*art26*	<	1 609 881–1 610 037	157
ABUWs029	*art27*	>	1 639 747–1 639 938	192
ABUWs030	*ffs* (SRP RNA)	>	1 745 277–1 745 406	130
ABUWs031	*art28*	>	1 754 539–1 754 654	116
ABUWs032	*art29*	>	1 763 969–1 764 059	91
ABUWs033	*art30*	>	1 773 546–1 773 770	225
ABUWs034	*art31*	>	1 811 325–1 811 466	142
ABUWs035	*art32*	>	1 913 010–1 913 201	192
ABUWs036	*art33*	>	1 963 079–1 963 258	180
ABUWs037	*art34*	<	2 042 167–2 042 562	396
ABUWs038	*art35*	>	2 047 846–2 048 195	350
ABUWs039	*art36*	<	2 130 793–2 130 882	90
ABUWs040	*art37*	<	2 261 072–2 261 224	153
ABUWs041	*art38*	<	2 299 341–2 299 425	85
ABUWs042	*art39*	>	2 431 101–2 431 291	191
ABUWs043	*art40*	<	2 431 080–2 431 299	220
ABUWs044	*art41*	<	2 510 733–2 510 999	267
ABUWs045	*art42*	>	2 544 369–2 544 483	115
ABUWs046	*art43*	<	2 590 856–2 591 043	188
ABUWs047	*art44*	<	2 626 894–2 627 090	197
ABUWs048	*art45*	<	2 684 695–2 684 881	187
ABUWs049	*art46*	<	2 686 551–2 686 764	214
ABUWs050	*art47*	<	2 687 414–2 687 573	160
ABUWs051	*art48*	<	2 687 680–2 687 885	206
ABUWs052	*art49*	<	2 824 022–2 824 378	357
ABUWs053	*ssrS* (6S RNA)	>	2 870 610–2 870 897	288
ABUWs054	*art50*	>	2 875 807–2 875 954	148
ABUWs055	*art51*	<	2 982 596–2 983 016	421
ABUWs056	*art52*	>	3 011 642–3 011 856	215
ABUWs057	*art53*	>	3 036 745–3 036 862	118
ABUWs058	*art54*	<	3 037 346–3 037 443	98
ABUWs059	*ssrA* (tmRNA)	<	3 041 416–3 041 910	495
ABUWs060	*art55*	>	3 051 590–3 051 768	179
ABUWs061	*art56*	<	3 057 837–3 057 964	128
ABUWs062	*rnpB*	<	3 109 445–3 109 818	374
ABUWs063	*art57*	>	3 121 389–3 121 542	154
ABUWs064	*art58*	>	3 127 099–3 127 277	179
ABUWs065	*art59*	<	3 214 805–3 214 860	56
ABUWs066	*art60*	<	3 223 892–3 224 279	388
ABUWs067	*art61*	<	3 224 468–3 224 548	81
ABUWs068	*art62*	<	3 224 584–3 224 664	81
ABUWs069	*art63*	<	3 224 700–3 224 778	79
ABUWs070	*art64*	>	3 311 767–3 311 902	136
ABUWs071	*art65*	<	3 368 843–3 369 267	425
ABUWs072	*art66*	>	3 389 780–3 390 024	245
ABUWs073	*art67*	<	3 389 980–3 390 179	200
ABUWs074	*art68*	<	3 495 799–3 495 971	173
ABUWs075	*art69*	>	3 524 369–3 524 892	524
ABUWs076	*art70*	<	3 570 390–3 570 549	160
ABUWs077	*art71*	<	3 570 844–3 570 997	154
ABUWs078	*art72*	>	3 621 445–3 621 557	113
ABUWs079	*art73*	>	3 677 044–3 677 147	104
ABUWs080	*art74*	>	3 682 160–3 682 299	140
ABUWs081	*art75*	>	3 766 134–3 766 270	137
ABUWs082	*art76*	<	3 783 241–3 783 311	71
ABUWs083	*art77*	>	3 863 958–3 864 104	147
ABUWs084	*art78*	>	3 958 954–3 959 174	221

### Transcript validation and false-positive assessment

**Fig. 1.**
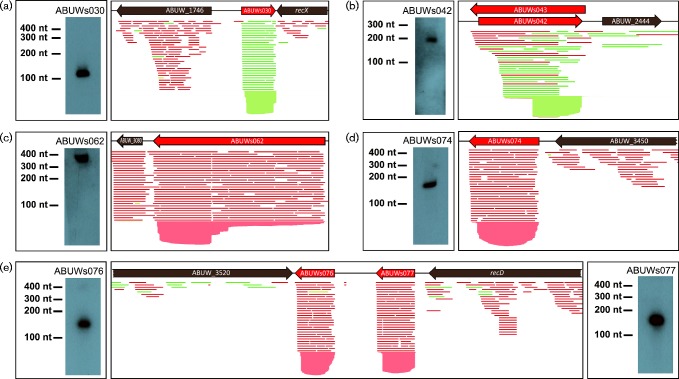
Northern blot detection of *A. baumannii* sRNAs. RNA was isolated from *A. baumannii* grown in LB for 3 h and subjected to Northern blot analysis. For sRNAs (a) ABUWs030, (b) ABUWs042, (c) ABUWs062, (d) ABUWs074, and (e) ABUWs076 and ABUWs077 the expected size from RNA-seq experiments was identical to that determined by Northern blot. Reads aligned to the reference genome are depicted in green (sense direction) or red (antisense); whilst existing annotations are highlighted in grey, and newly annotated sRNAs are in red.

In order to validate our RNA-seq data, Northern blotting was used to confirm the existence of the identified Art transcripts, as well as their size and abundance. As a proof of principle, for six of the most abundant sRNAs, we designed transcript-specific probes and were able to detect bands of the expected size (Fig. 1). To minimize the possibility of falsely annotating protein-coding genes as sRNAs we next performed a search for potential ORF-containing sequences in each of the transcripts. For 19 of the Arts we detected one or more possible ORFs of ≥ 30 aa (Table S2). Eleven of these sequences showed no significant similarity to other known proteins, whilst the remaining seven showed similarity (*E*-value ≤ 1 × 10^− 10^) only to hypothetical proteins present in a handful of *A. baumannii* strains. For these putative proteins, no functional description exists nor do they possess any apparent functional domains. As such, the probability that the putative ORFs within the *art* sequences specify legitimate proteins is rather low and therefore possibly represents incorrectly ascribed protein-coding genes. Nevertheless, the possibility of coding functions for these transcripts cannot be completely excluded, particularly as a number of sRNAs presumed to be strictly non-coding have been recently shown to in fact specify small peptides with functional roles (Andrews & Rothnagel, 2014; Balasubramanian & Vanderpool, 2013; Gimpel *et al.*, 2010; Hobbs *et al.*, 2011). Furthermore, one of the newly annotated *A. baumannii* sRNA genes, ABUWs055, overlaps with the previously identified gene ABUW_2969. As a result, ABUWs055 potentially includes part of the protein-coding sequence of ABUW_2969 in its ORF. In disagreement with this, however, is the observation that no ribosome-binding site is present for the internally transcribed region, making a protein-coding function unlikely; pointing instead towards a role as a regulatory RNA.

### sRNAs are amongst the most highly expressed genes in *A. baumannii*

**Fig. 2.**
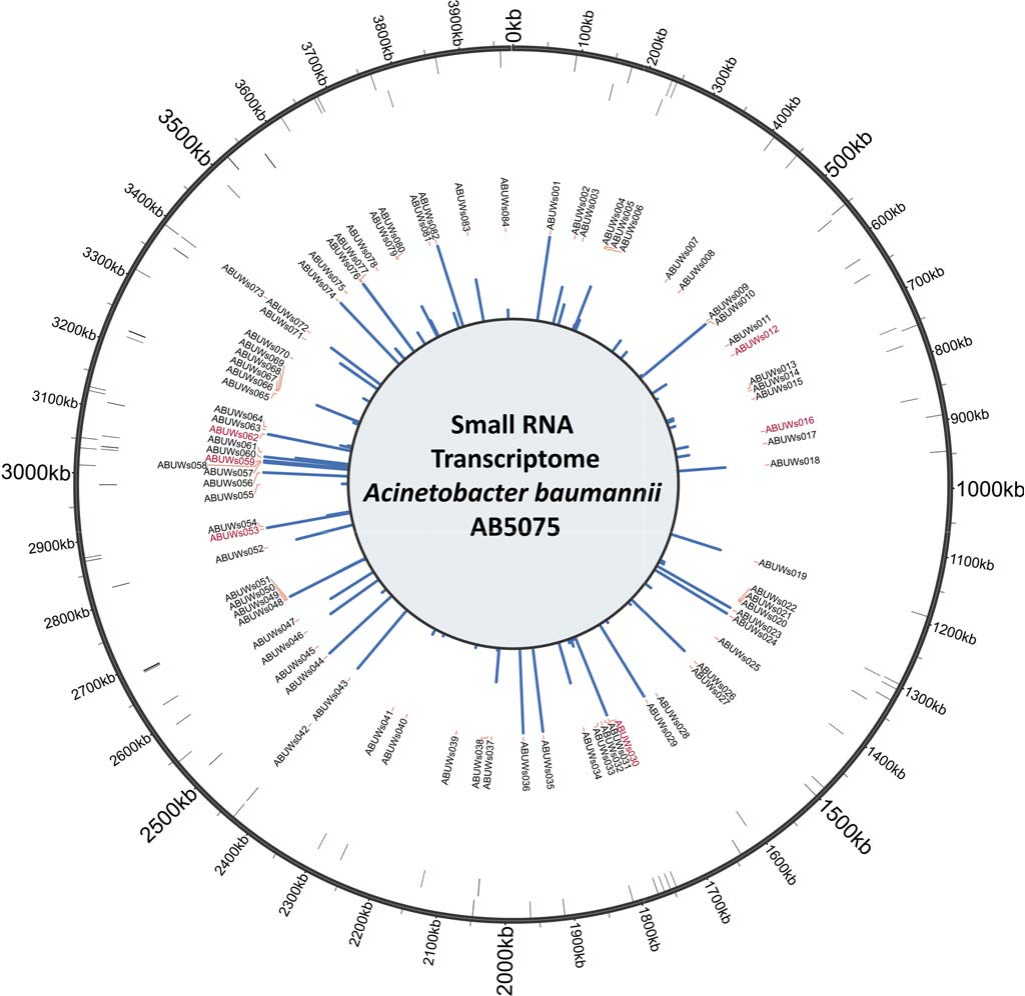
Transcriptional activity and genomic context of newly identified *A. baumannii* sRNAs. RNA-seq was performed with RNA isolated from *A. baumannii* cultures grown for 3 h in LB. The outer circle marks the position of sRNAs within the genome. The second and third circles highlight the location of novel sRNAs on the forward or reverse strand, respectively. The transcript designations are depicted above the inner circle (novel transcripts in black and previously described transcripts in red), which indicates expression values for each sRNA (maximum displayed expression value: 2000).

To test the functional application of our newly created, sRNA-containing annotation file, as well as to gain more insight into sRNA transcript abundance, we used the RNA-seq dataset to generate expression values for each of the sRNA genes (Table S3). We found the known RNA species SRP RNA, 6S RNA and tmRNA (ABUWs030, ABUWs053 and ABUWs059, respectively) to be very highly expressed, representing three of the five most highly transcribed genes in the entire genome (after rRNA depletion). Moreover, half of the 20 most abundant transcripts expressed in AB5075 were in fact sRNAs. Even more interestingly, under the tested conditions, the newly annotated sRNAs constituted ∼27 % of all reads that align to any region annotated as a gene (excluding rRNA genes), whilst at the same time they represented only ∼2 % of all annotations. The location, orientation and expression value for each annotated sRNA are presented in Fig. 2. These findings not only emphasize the presence of several highly expressed sRNAs within the genome, but also point to potential physiological relevance that might be expected for such strongly expressed genes.

### Conservation of AB5075 sRNAs across diverse *Acinetobacter* strains and species

**Fig. 3.**
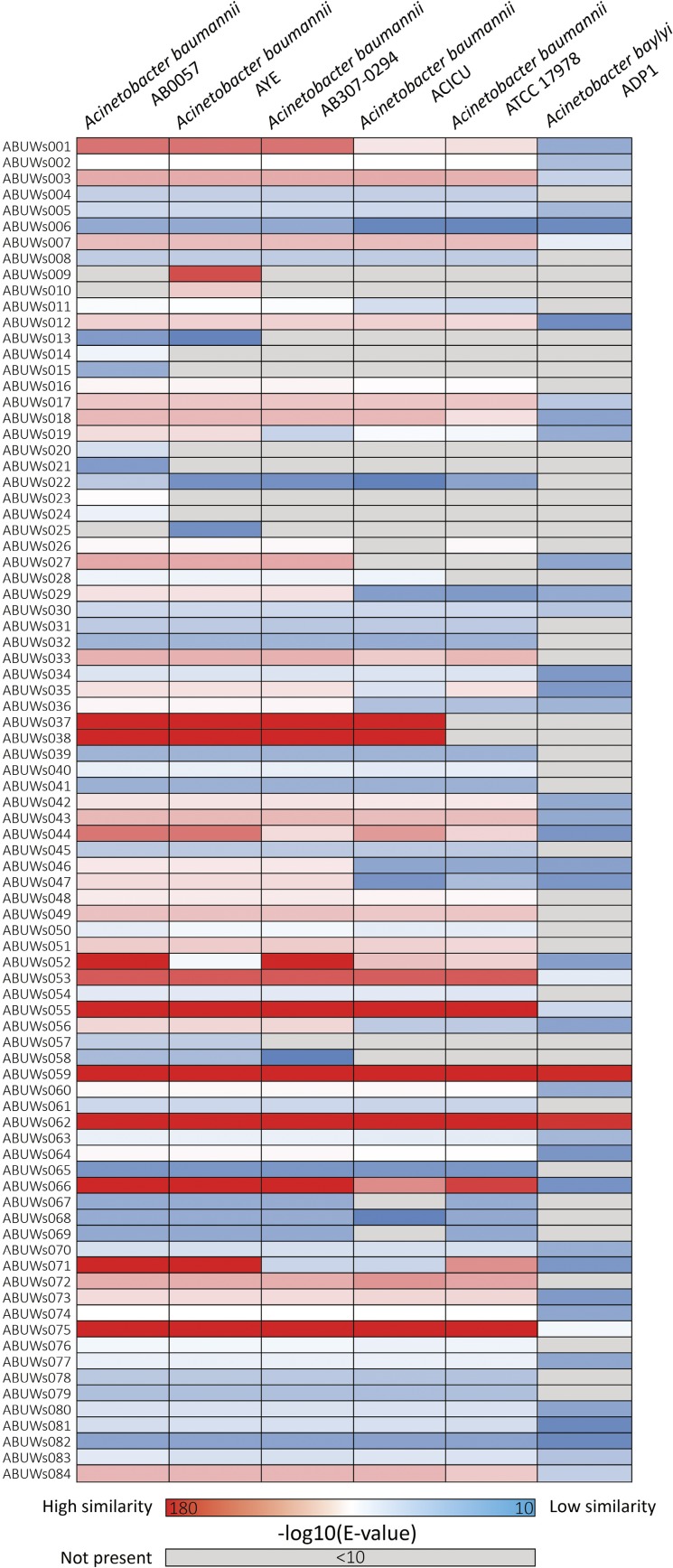
Conservation of *A. baumannii* AB5075 sRNAs in other *Acinetobacter* strains and species. Shown are the presence and conservation of AB5075 sRNAs in other *Acinetobacter* strains (*A. baumannii* AB0057, AYE, AB307-0294 and ATCC 17978) and species (*A. baylyi* ADP1). Data are presented in the form of a heatmap, generated using negative log-transformation of *E*-values received from a nucleotide blast of each AB5075 transcript against relevant genomes. The lowest *E*-value returned is displayed for each search of an sRNA against a given genome.

Having identified and confirmed the annotation of sRNA transcripts in strain AB5075, we next investigated whether the corresponding genes/genomic regions are also present in other *A. baumannii* strains or *Acinetobacter* spp. This is particularly relevant as AB5075 is a relatively new and highly virulent strain that differs from its less pathogenic counterparts and traditionally employed laboratory strains (Jacobs *et al.*, 2014). To do this we chose to compare the AB5075 sRNA content to the historically investigated *A. baumannii* strain ATCC 17978, as well as more recently isolated clinical variants (AB0057, AYE, AB307-0294 and ACICU), and the naturally transformable (Juni & Janik, 1969) and non-pathogenic *A. baylyi* strain ADP1 (Barbe *et al.*, 2004). Following blast analysis, *E*-values were subject to negative log-transformation and visualized as a heatmap (Fig. 3). A small number of sRNAs showed very high levels of conservation amongst all strains, including the evolutionarily more distant *A. baylyi* strain. As expected, the widely distributed tmRNA (ABUWs059) and RNase P RNA (ABUWs062) showed strong conservation. Additionally, several other transcripts, such as ABUWs066, ABUWs075 and ABUWs084, were conserved in all strains investigated, suggesting a basic and fundamental physiological role in *Acinetobacter* spp. Conversely, many other transcripts are only present either in a small subgroup of strains or almost exclusively in AB5075. This is particularly true for ABUWs009 and ABUWs010, which are only present in *A. baumannii* AB5075 and AYE. Similarly, ABUWs014, ABUWs015, ABUWs020 and ABUWs023 are found only in strains AB5075 and AB0057. Another subgroup of sRNAs (i.e. ABUWs033, ABUWs040, ABUWs045, ABUWs065 and ABUWs076) is confined to only *A. baumannii* and is entirely absent in *A. baylyi*. Although this approach only confirms the presence/absence of sRNA genes, and does not confirm actual expression in other strains/species, it does provide a strong foundation for future sRNA conservation studies in the genus *Acinetobacter*.

One of the most interesting findings amongst the newly identified transcripts concerns ABUWs037 and ABUWs038; the latter is antisense to the *acdP* gene whilst ABUWs037 is a standalone transcript. These sRNAs are part of an almost 100 kb region (flanked by genes *adeB* and ABUW_2084) that demonstrates significant genomic variability. Specifically, large portions of this region are seemingly entirely absent in the ATCC 17978 strain, including the ABUWs037 and ABUWs038 transcripts. Such an observation highlights the genomic flexibility of *A. baumannii* and demonstrates that differences occur, not only for protein-coding regions, but also in sRNA content. This kind of event may explain the differences observed amongst isolates and potentially is causative for the enhanced virulence of recently emerged clinical strains.

### Several families of *A. baumannii* sRNAs exist that exhibit structural similarities and modular organization

Whilst performing blast analyses, comparing sRNAs annotated in AB5075 to other strains and species, we noted that a large number of searches resulted in multiple hits within each genome. To understand this observation, we performed a ‘self-blast’ of the AB5075 sRNAs directly with the AB5075 genome, finding that a large number of homologous regions exist for several of the annotated sRNA genes. Specifically, we identified six groups of sRNAs based on internal homology (Table 2). Notably, the ‘self-blast’ analysis only identified sRNAs already annotated as Art transcripts in this study, whereas none of the blast results revealed similarity to an area of the genome that was not expressed or already annotated as an sRNA herein. As such, each element within the six families identified is a *bona fide* expressed transcript. This is a remarkable observation, as it corroborates our method of sRNA identification and presents an entirely new finding of high sequence similarity for a large number of sRNA transcripts in *A. baumannii*. To investigate the extent to which these sequence similarities transfer into structural relatedness, we performed alignments for each of the groups and identified domains of high conservation. Subsequently, we generated structural predictions for each of the transcripts, highlighting conserved regions and comparing individual structures with one another.

**Table 2.** Grouping of *A. baumannii* AB5075 sRNAs according to sequence similarity

Group 1	Group 2 (antisense to Group 1)	Group 3	Group 4	Group 5	Group 6
ABUWs001	ABUWs043	ABUWs005	ABUWs013	ABUWs041	ABUWs032
ABUWs006*	ABUWs072	ABUWs061	ABUWs021	ABUWs048	ABUWs050
ABUWs018		ABUWs081			
ABUWs019		ABUWs083			
ABUWs026					
ABUWs029					
ABUWs035					
ABUWs036					
ABUWs042					
ABUWs044					
ABUWs046					
ABUWs047					
ABUWs052					
ABUWs056					
ABUWs058*					
ABUWs066*					
ABUWs067*					
ABUWs068*					
ABUWs069*					
ABUWs071					
ABUWs073					

* Truncated genes that do not contain Region 1.

### Group 1: a collection of highly conserved, phage-derived transcripts

The largest group of homologous sRNAs identified harboured 21 similar sequences. An alignment of these genes revealed three strongly conserved domains, referred to as Regions 1, 2 and 3 (Fig. S1); although six of the genes have a truncation at their 5′ end that results in the loss of Region 1. Despite Regions 2 and 3 being conserved across all sRNAs in the group (including the six truncated transcripts), we focused our further analysis only on the 15 full-length sRNAs.

**Fig. 4.**
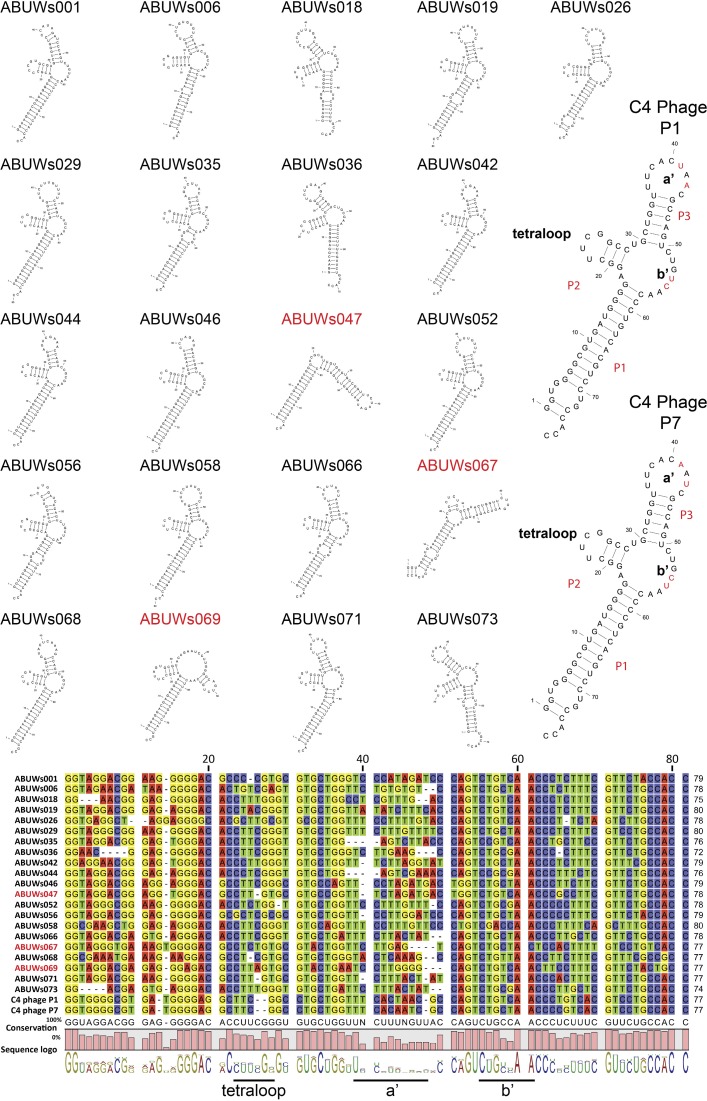
Group 1 sRNAs are a family of highly conserved, phage-derived transcripts. Regions 2 and 3 from Group 1 sRNAs were isolated and an alignment created, uncovering strong similarity to the C4 RNA of phages P1 and P7. Folding predictions demonstrated that all but three transcripts (ABUWs047, ABUWs067 and ABUWs069; highlighted in red) show characteristic structural features of the C4 molecule; consisting of three stems and three loops (termed the tetraloop, loop a′ and loop b′). For the phage molecules, divergent residues between P1 and P7 are shown in red.

Three secondary structure prediction algorithms (CLC, mfold and RNAfold) were used to analyse these sRNAs and, despite minor variability in overall folding, each of the three regions displayed high structural similarity, adopting only a limited number of conformations (Fig. S2). Region 1 almost exclusively forms a stem–loop of 6 or 7 nt with a loop consensus motif of CACAUU or CUACAUU. Although more variation was observed in the structural predictions for Regions 2 and 3, a conserved motif was identified as an interaction between the two regions. This interaction becomes even more apparent when, instead of using full-length transcripts, only Regions 2 and 3 are folded together independently (Fig. 4). When using the Rfam secondary structural motif search (Nawrocki *et al.*, 2015) we found that the interaction of Regions 2 and 3 is in fact a conserved feature found in bacteriophages P1 and P7 (GenBank accession numbers AF234172 and AF503408), termed the C4 antisense motif. The phage C4 antisense RNA is a critical component of a complex immunity system for phages, which prevents superinfection, and promotes the establishment and maintenance of lysogeny within host bacteria (Citron & Schuster, 1990, 1992; Heinrich *et al.*, 1995). In this situation, the C4 antisense RNA maintains a distinct secondary structure, characterized by three stem–loops (P1, P2 and P3; Fig. 4). Additionally, the C4 antisense RNA is itself processed from a larger transcript to a ∼77 ± 1 bp active form by host RNase P at the 5′ end of the stem of the P1 hairpin (Hartmann *et al.*, 1995). After processing, C4 loops a′ and b′ interact with target mRNAs, such that the b′ loop partially occludes ribosome-binding sites, with the a′ loop providing target specificity by binding to upstream sequences. It is noteworthy that the only region of variability in the alignment of Group 1 sRNAs with the P1 and P7 C4 molecules is in the target-specificity-mediating a′ loop (7–11 bp). As such, if the C4 mode of interaction is maintained in *A. baumannii*, this could allow these sRNAs to bind to a variety of different target RNA sequences.

**Fig. 5.**
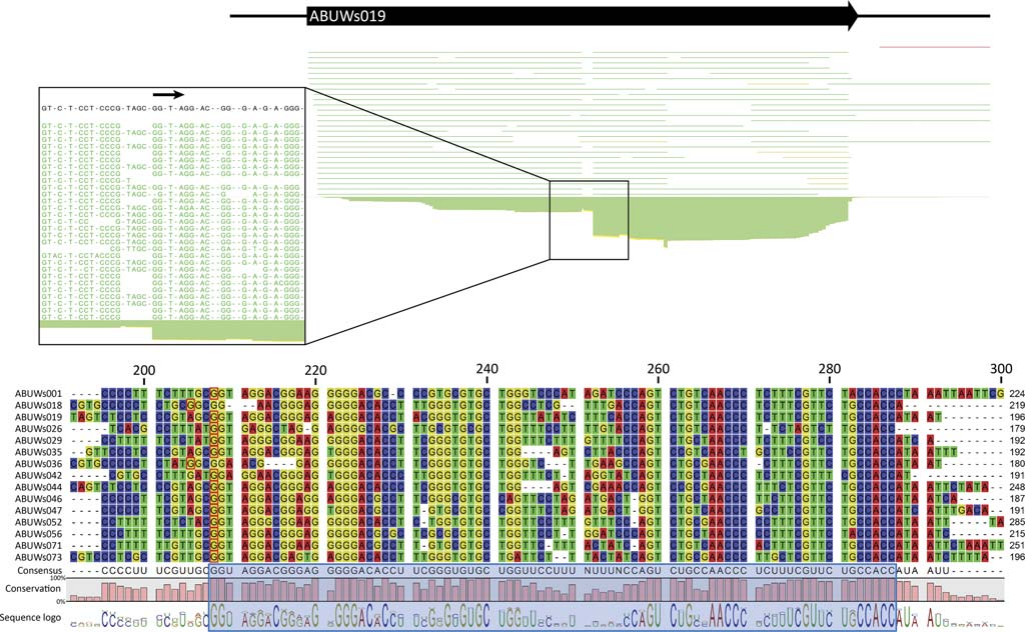
*A. baumannii* Group 1 transcripts appear to be processed in a manner akin to that observed for C4. RNA-seq reads aligned to Group 1 sRNAs were investigated for differences in coverage between the 5′ end and the C4 region (blue box). sRNA ABUWs019 was chosen as a representative example, highlighting that the region homologous to C4 shows significantly higher sequence coverage than the upstream portion of the transcript. Interestingly, this region of high coverage begins with a typical GGW motif (marked by a black arrow), which mirrors the RNase P processing site (GGT) for phage C4 antisense transcripts. The putative GGT or GGA cleavage sites (the first G is highlighted for each sRNA by a red box within the alignment) was found for all but two of the transcripts (ABUWs018 and ABUWs036). These latter elements appear to be processed 3 nt earlier, at a GGC motif. Alignments of RNA-seq reads for the remaining Group 1 members, showing putative processing sites, can be found in Fig. S3.

To explore whether Regions 2 and 3 were in fact processed from their larger transcript, as seen for the C4 region in phages P1 and P7, we analysed our RNA-seq dataset for distinct expression patterns within the different Group 1 sRNAs. For every Group 1 transcript that possessed an extended 5′ region (full-length transcripts that contain Region 1), we observed far more reads for the regions homologous to C4 (Regions 2 and 3) than for the upstream transcript (Region 1) (an example is presented in Fig. 5; for other Group 1 sRNAs, refer to Fig. S3). Furthermore, for all but two of the transcripts, the area of higher read coverage began with a characteristic GGW motif (Fig. 5), which has previously been identified as the RNase P processing site for phage P1 and P7 C4 antisense RNAs (Hartmann *et al.*, 1995). Even in the two molecules that did not retain this processing pattern (ABUWs018 and ABUWs036), a GGC sequence was observed at the start of the putatively processed region, suggesting perhaps that only the GG motif is required for recognition and processing by RNase P in *A. baumannii*.

Interestingly, despite strong conservation amongst the sRNA transcripts in Group 1, Region 1 displayed very limited sequence homology with upstream sequences in the P1 and P7 phage genomes. Therefore, it is possible that this motif was duplicated alongside the original C4 transcript following an initial integration event and perhaps serves an auxiliary function to the core C4 motif.

### Group 2: putative antisense regulators of Group 1 sRNAs

**Fig. 6.**
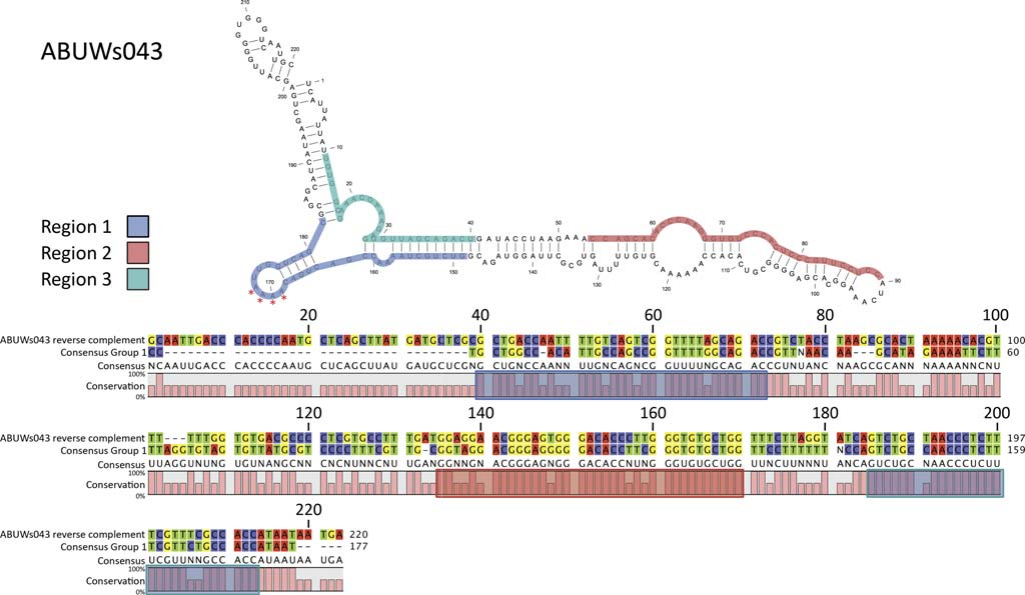
Group 2 sRNAs encode potential Group 1 antisense transcripts. Shown is an alignment for reverse and complemented ABUWs043 against the consensus sequence of Group 1 sRNAs (from alignment in Fig. S1). Structural predictions for ABUWs043 reveals a potential area of initial interaction with Group 1 transcripts via a series of exposed U residues (shown on the figure as red stars). These could potentially partner with an accessible region of exposed A nucleotides within members of Group 1 (marked with red arrows in Fig. S2).

The two members of group 2, ABUWs043 and ABUWs072, are also of significant interest as both are located antisense to a member of Group 1: ABUWs043 is antisense to ABUWs042 (Fig. 1b) and ABUWs072 partially overlaps antisense to the 3′ region of ABUWs071. The reverse and complemented sequence for both Group 2 sRNAs showed high similarity to the consensus sequence of Group 1 elements (Fig. S4), with ABUWs043 possessing the greatest homology: having sequence identities of 79, 83 and 90 %, respectively, for Regions 1, 2 and 3 (Fig. 6). This conservation presents the possibility that Group 2 sRNAs are able to interact in an antisense fashion over long stretches with different Group 1 transcripts, which would allow them to affect several targets, rather than just one (Gottesman & Storz, 2011; Thomason & Storz, 2010). Structural predictions for ABUWs043 revealed a loop at nt 170 that has an exposed 4A stretch, which could possibly interact with a 4U counterpart in Group 1 molecules (Fig. 6). Such a corresponding sequence was largely conserved and accessible in the structures of Group 1 sRNAs (demarcated by red arrows in Fig. S2). It should be noted that, given the relatively low abundance of both Group 2 transcripts, particularly in comparison with their Group 1 counterparts, Northern blot detection of their presence in the cell was not possible. Nevertheless, although there were only two Group 2 elements, these sRNAs have the potential to couple with a variety of targets within the *A. baumannii* cell, suggesting that they could be inordinately powerful regulators of sRNA activity.

### Groups 3–6: recurring sRNA structures and modularity

**Fig. 7.**
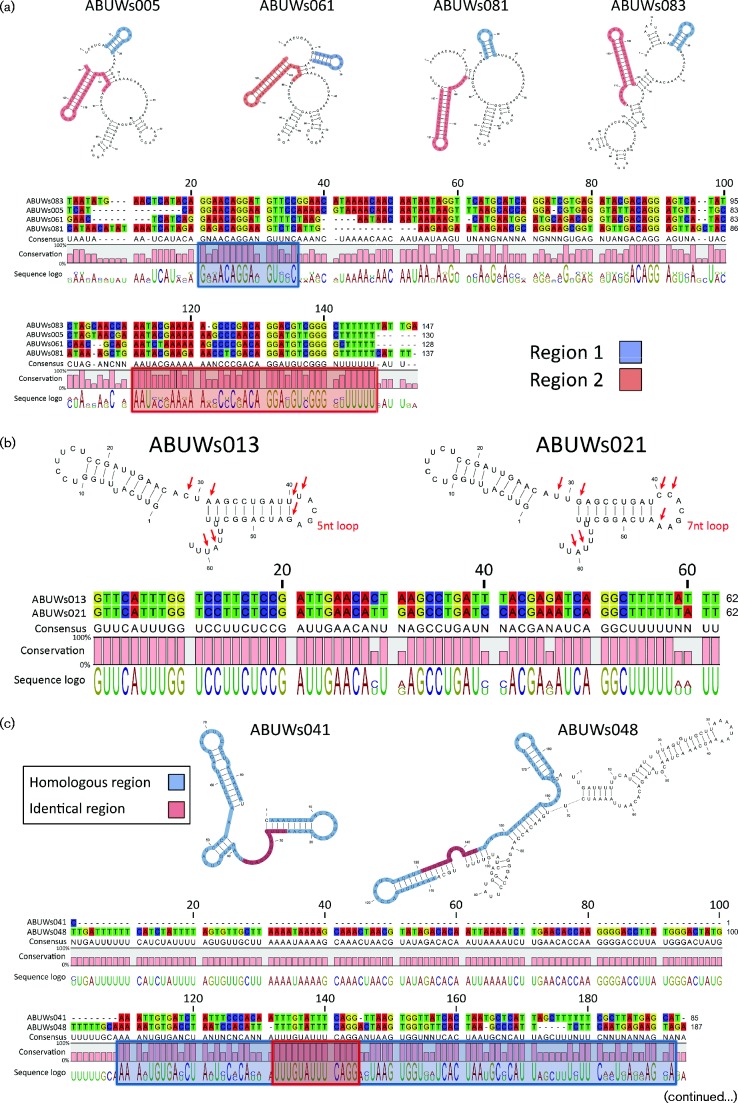

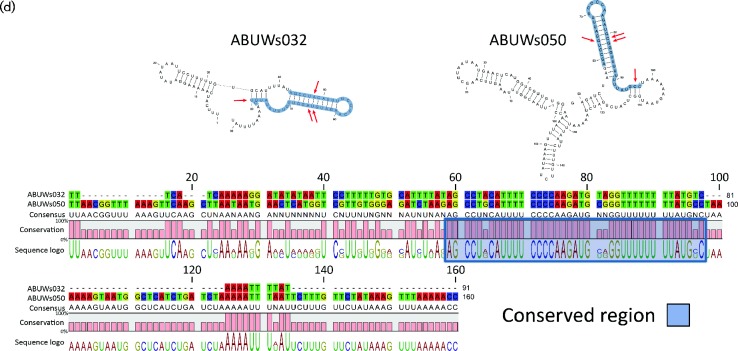
*A. baumannii* sRNAs from Groups 3–6 maintain discrete structural relatedness and a modular organization. sRNAs with highly conserved sequences were grouped and their secondary structures predicted using the CLC Genomics Workbench software. Conserved structures within each group are highlighted. Red arrows in panels (b) and (d) indicate residues of clear sequence divergence or similarity.

With four homologous transcripts, Group 3 is the second largest collection of sRNAs in *A. baumannii* AB5075. Analogous to Group 1, all members display multiple regions of structural conservation (Fig. 7a). For all four transcripts, Region 1 forms a short hairpin with a largely conserved loop sequence, whilst Region 2 forms an extended hairpin. Beyond the two conserved regions, all four members of the group display two largely open structures as well as two or three additional hairpins.

Group 4 contains two members (Fig. 7b), ABUWs013 and ABUWs021, which are almost entirely identical (89 % sequence identity). Both appear to form two hairpins, with the 5′ example being completely conserved between the two structures. Differences between these highly homologous sRNAs are largely confined to the region connecting the hairpins, within the loop of the second hairpin and in the unbound 3′ region of the transcripts. Interestingly, alterations in hairpin 2 result in different loop lengths of 5 and 7 nt for ABUWs013 and ABUWs021, respectively.

The two members of Group 5, ABUWs041 and ABUWs048, display significantly different sizes of 85 and 187 nt, respectively. Despite this, alignment of their sequences reveals a high level of similarity for ABUWs041 to the 3′ end of ABUWs048 (Fig. 7c), with a stretch of 13 identical residues occurring in the middle of a region of high conservation within both transcripts. Structural predictions for both sRNAs demonstrate that the overall conserved area includes two (ABUWs048) or three (ABUWs041) hairpins and that in both structures the most conserved region harbours an accessible UUUC sequence. Other than these two features, ABUWs048 has two additional hairpin structures, demonstrating how the modular arrangement of sRNAs can essentially be extended *ad libitum*.

Group 6 similarly consists of two transcripts that possess a variety of distinct structures. Whilst ABUWs032 harbours two hairpins, ABUWs050 consists of three extended hairpin structures as well as one shorter hairpin (Fig. 7d). The homologous region for both transcripts contains the larger hairpin of each molecule, which displays only 4 nt differences between the two sequences. Interestingly, two of these differences are a pairing couple within the stem–loop, indicating coordinated mutation or evolution of sequence. A further nucleotide variation is immediately adjacent to this, but comes with no concomitant partner mutation, at least in ABUWs050, as alternative pairing (i.e. wobble) accommodates the base change. In this way, the loop region, as well as the stem structure, is largely similar between the two transcripts. Only the long uracil stretches within the stem generates an open structure in ABUWs032, whilst the corresponding area in ABUWs050 maintains interaction with the opposite side of the stem.

### Conservation of the *A. baumannii* sRNA Families

With regard to the conservation of the six groups amongst *A. baumannii* strains beyond AB5075, most transcripts from Groups 3–6 show similarity to those from other isolates included in this study, with the exception of Group 4. Curiously, Group 4 sRNAs, and particularly ABUWs021, display very limited similarity to any genomic region of the other *A. baumannii* strains (apart from AB0057), therefore potentially contributing to AB5075-specific physiology and virulence. Comparisons with other bacterial species outside of the genus *Acinetobacter* using a nucleotide blast analysis indicates that no sequence homologues for Group 3–6 structures are present within other organisms (no significant hits of *E*-value ≤ 1 × 10^− 15^). Due to their restricted presence in other bacterial species, it is perhaps unsurprising that no information about the function of transcripts from these groups can be assigned by comparison to already described sRNAs or sRNA structural features (e.g. Rfam). Whilst the conservation of sRNAs in Groups 3–6 is relatively clear, the presence or absence of Group 1 and 2 homologues is not so easily evaluated. Due to the high similarity of Group 1 transcripts to each other, and also to the antisense sRNAs of Group 2, one cannot readily, and specifically, distinguish between the various transcripts using blast analyses. It is, however, possible to confirm that each of the Group 1 *A. baumannii* AB5075 sRNAs show similarity to a large number of sRNAs in all of the other *A. baumannii* strains assessed herein. Thus, whilst the specific conservation of individual elements cannot be categorically affirmed, it is apparent that various Group 1 transcripts are present, and broadly conserved, across *A. baumannii* isolates. Therefore the putative C4 antisense RNAs are seemingly a common theme in this organism, suggesting a retained and central role in species-specific behaviour, and, potentially, virulence.

## Discussion

Recent advances in both RNA-seq technology and sophisticated, bioinformatic transcript-prediction algorithms have facilitated the rapid and reliable identification of novel RNA and sRNA transcripts (Backofen & Hess, 2010; Livny & Waldor, 2007; Pichon & Felden, 2008; Sharma & Vogel, 2014). These advantages, however, have not been without drawbacks, as the integration of data between RNA-seq and computational datasets is often problematic. Specifically, erroneous annotations in automated genome annotation approaches complicate and confound the integration of newly identified transcripts into existing genome files (Sridhar & Gunasekaran, 2013).

One commonly encountered challenge in this regard is the validity of ‘hypothetical proteins’ identified by computational means, particularly when these only partially overlap with RNA-seq reads. For example, in the present study, one hypothetical protein-coding transcript, ABUW_0220, showed similarity to several of the newly annotated sRNAs from Group 1. A blast search revealed that the putatively 70 aa ABUW_0220 protein does not possess any known functional domains and, furthermore, shows similarity only to hypothetical proteins from other *A. baumannii* strains. When one looks at RNA-seq alignments for this gene, it becomes clear that mRNA reads only cover the 5′ region of this hypothetical protein-coding region and do not extend to the predicted translational stop codon (Fig. S5a). Instead, reads not only map to the predicted confines of a putative Group 1 sRNA, but a clear region of higher coverage, as seen for the processing sites of other Group 1 sRNAs, is readily apparent. Moreover, the putatively processed region of this transcript perfectly aligns with the 5′ and 3′ ends of the processed C4-derived transcripts in Fig. S5(b). As such, due to the lack of similarity to other known proteins, the short length of the hypothetically encoded protein sequence, and the retained sequence homology and putative processing patterns with Group 1 sRNA transcripts, it cannot be excluded that ABUW_0220 is in fact a misannotated sRNA, rather than an actual protein-coding gene. However, despite the evidence that ABUW_0220 could indeed be an sRNA gene, exclusion of this hypothetical protein from genome annotation files could hinder future studies, should a corresponding polypeptide product be identified. Therefore, for this study, we elected not to include the annotation of this locus as an sRNA.

A comparison of RNA-seq datasets across different strains of the same bacterial species can be equally problematic. For example, although we found the previously identified sRNA AbsR28 was expressed in our RNA-seq experiment, two other sRNA genes that were experimentally identified in the same study by Sharma *et al.* (2014) either completely (AbsR25, overlapping with ABUW_2553) or largely (AbsR11, overlapping with the 5′ region of *cyoA*) overlapped with previously annotated and protein-coding genes in the AB5075 background. As the study identifying these transcripts was performed in a different *A. baumannii* background (MTCC1425), strain-specific differences cannot be excluded, thereby necessitating a conservative approach in which we did not add the two genes to our genome annotation file.

The evaluation of computationally identified sRNAs is a complicating factor, as they may either be misidentified and not exist within the cell, or be valid transcripts but not detected due to strain- and growth-specific conditions. These predictions are thus best understood as guides for investigation, rather than *de facto* genes, requiring secondary experimental validation. In the context of this study, 28 hypothetical sRNA genes bioinformatically predicted by Sharma *et al.* (2014) produced no transcriptional activity in our experiments. As we have no validation for their existence, we have again taken the conservative approach of omitting them from our annotation file.

Regardless of these challenges, it is nonetheless of importance that we gather comprehensive and complete data about the small RNome from a wide variety of bacteria. Such data facilitates the cross-comparison of small transcripts between different species, as has been used countless times for the study of proteins, and provides insight into the conservation of these elements across different branches of life. In addition to the identification and evaluation of undescribed sRNAs, we contend that it is important to establish annotation, nomenclature and cataloguing systems that can be maintained and extended to accommodate future studies. The systematic addition of sRNAs to existing annotation files is the cornerstone of such a process, promoting differentiation between sRNAs that are either (1) conserved amongst a wide number of strains or (2) specific to virulent (or avirulent) lineages. In this regard, our analysis is an initial, but key, step towards the complete identification of sRNAs in the important human pathogen *A. baumannii*.

Analysis of the internal conservation of sRNA elements in *A. baumannii* AB5075 in this study resulted in the discovery of a number of short, conserved sequences present in multiple sRNA transcripts. These sequences, when studied by themselves, out of context of the full-length transcript, adopt very limited numbers of conformations (usually only one). Thus, they may represent modules of functional conservation that have been interspersed with variable regions and/or arranged in varying combinations with other modules. This modularity may be comparable to the use of conserved functional units within proteins (Peterman *et al.*, 2014), such as DNA-binding or kinase domains, which appear in variable combinations with other domains to produce unique structural and functional products. Therefore, with continued advances in the annotation of conserved sRNA sequences across bacterial genera, the identification of core structural sRNA elements may allow for the rapid prediction of mechanisms of action for novel sRNAs based on these traits. Indeed, this concept has been suggested for the different functional modules of the MicA sRNA in *Escherichia coli*, where distinct structural domains of the transcript separately affect stability and target specificity (Andrade *et al.*, 2013).

From a function perspective, conserved structural domains often establish the basic mechanisms of action for a given molecule, whilst variable domains (e.g. the a′ and b′ loops of the C4 antisense RNA) are associated with target specificity. As such, the C4-derived transcripts may (1) target incoming bacteriophage and/or (2) interact with bacterial transcripts to post-transcriptionally regulate their expression. With regard to the former assertion, C4 antisense RNAs that originally targeted phage P1 and P7 genomic sequences may have integrated into the genome of *A. baumannii*, and related bacteria, as a mechanism to prevent (super)infection with phages P1, P7 and other related viruses that utilize C4 antisense RNA systems. The second, and potentially more likely, role for the C4-derived sRNAs may be their adaptation to target bacterial RNA transcripts. This contention is strengthened by the observation that the sRNAs of Group 1 show significant differences in the a′ region, which has been shown to mediate target specificity (Citron & Schuster, 1990). Indeed, a deviation in this region of only 4 nt within the C4 transcript and corresponding targets from bacteriophages P1 and P7 is sufficient to restrict cross-reactivity between target and effector pairs (Citron & Schuster, 1990). Consequently, the alterations observed in the a′ and b′ regions of *A. baumannii* Group 1 sRNAs may have evolved to affect a number of different bacterial RNAs.

Despite the large abundance of bacteriophages in nature, to the best of our knowledge this is the first report of a large group of bacterial sRNAs within a single genome that may have co-opted functional secondary RNA structure in this way. Moreover, this adaptation of phage-derived RNA secondary structure toward novel targets may not be isolated, as Weinberg *et al.* (2010) bioinformatically predicted the C4 antisense RNA motif in a number of other bacteria, including the *Burkholderiales*, *Enterobacteriales*, *Pasteurellales*, *Pseudomonadales*, *Vibrionales*, *Xanthomonadales* and *Caudovirales* (Weinberg *et al.*, 2010). We experimentally elaborate on these works, demonstrating that the predicted structural elements are in fact highly expressed and appear to be processed from a larger transcript, further adding weight to the notion that they have actual physiological importance, at least within *A. baumannii* cells.

Although the C4-like sRNAs are compelling examples, they likely represent only the tip of the iceberg in terms of complexity, and extent of sequence and structural conservation for sRNAs. For example, evaluation of Group 2 sRNAs herein demonstrates their similarity to a large number of other transcripts (the Group 1 sRNAs), potentially allowing a rather small number of (antisense) sRNAs to have magnified regulatory impacts. This additional layer of control presents an elegant and efficient method for quickly altering cellular physiology through sRNA–sRNA interaction. Lastly, the presence of repetitive sRNA elements in a single genome (e.g. sRNA members of Groups 3–6), as well as the conservation of these elements, may provide insight into species-specific evolution. This is highlighted by the finding that certain groups (e.g. Group 4) are seemingly confined to the AB5075 background and display only limited similarity to other *A. baumannii* genomes. This information is of particular importance in determining how novel, highly virulent strains develop and how sRNAs contribute to bacterial pathogenicity. Collectively, our work has the potential to aid in the development of strategies to counteract the concerning and ever-increasing appearance of extremely pathogenic and antibiotic-resistant bacteria, which are amongst the most pressing of human challenges in the twenty-first century.

## Supplementary Data

11167 Supplementary Data
