# Supplementary material for: Towards the complete small RNome of Acinetobacter baumannii
Source: Microb Genom. 2016 Mar 2;2(3):e000045. doi: 10.1099/mgen.0.000045 (PMC5320573; doi:10.1099/mgen.0.000045)
Supplement: Supplementary file 1 — Supplementary Data [file mgen-02-45-s001.pdf]

Table S1: Genomic context of *Acinetobacter baumannii* AB5075 sRNAs

AB5075 designation	Genomic context	Upstream gene	Locus orientation	Downstream gene
ABUWs001	IG	*murI1*	> > >	ABUW_0085
ABUWs002	IG	ABUW_0140	< < <	*xth*
ABUWs003	IG*	ABUW_0157	> > >	ABUW_0158
ABUWs004	IG	ABUW_0207	> < >	ABUW_0208
ABUWs005	IG	ABUW_0211	< > <	ABUW_2012
ABUWs006	IG	ABUW_0219	< > >	ABUW_0220
ABUWs007	IG	*suhB*	> > >	ABUW_0381
ABUWs008	IG	ABUW_0436	> > >	ABUW_0437
ABUWs009	AS^{P} (ABUW_0545)	ABUW_0545	< > <	ABUW_0546
ABUWs010	IG	ABUW_0552	< < >	ABUW_0553
ABUWs011	OL^{P}* (ABUW_0627)	ABUW_0627	< < <	ABUW_0628
ABUWs012	IG	ABUW_0651	< < <	*lysP*
ABUWs013	AS^{P} (ABUWs0749)	ABUW_0749	> < >	ABUW_0750
ABUWs014	IG	ABUW_0764	> > >	ABUW_0765
ABUWs015	IG	ABUW_0801	> < >	ABUW_0802
ABUWs016	IG	ABUW_0880	> < <	*trpS*
ABUWs017	AS^{P} (*carA*)	*carA*	< > >	ABUW_0895
ABUWs018	IG	ABUW_0937	< > <	ABUW_0938
ABUWs019	IG	ABUW_1162	< > >	ABUW_1163
ABUWs020	IG	ABUW_1263	< > <	ABUW_1264
ABUWs021	AS^{P} (ABUW_1267 and ABUW_1268)	ABUW_1267	> < >	ABUW_1268
ABUWs022	IG	ABUW_1284	> > >	ABUW_1285
ABUWs023	IG	ABUW_1310	> > >	ABUW_1311
ABUWs024	IG	ABUW_1327	< < >	ABUW_1328
ABUWs025	AS^{P} (ABUW_1402)	ABUW_1402	> < >	ABUW_1403
ABUWs026	IG	ABUW_1497	< > >	ABUW_1498
ABUWs027	OL^{P}* (ABUW_1514)	ABUW_1513	> > >	ABUW_1514
ABUWs028	OL^{P} (ABUW_1616)	*glnS*	> < <	ABUW_1616
ABUWs029	IG	ABUW_1644	< > >	ABUW_1645
ABUWs030	IG	ABUW_1746	< > <	*recX*
ABUWs031	IG	ABUW_1758	> > >	ABUW_1759
ABUWs032	IG	ABUW_1764	< > <	mobile element
ABUWs033	AS (ABUW_1773)	ABUW_1773	< > >	ABUW_1774
ABUWs034	IG	ABUW_1815	> > >	*aro1*
ABUWs035	IG	ABUW_1910	< > <	ABUW_1911
ABUWs036	IG	ABUW_1958	> > >	ABUW_1959
ABUWs037	IG	ABUW_2060	< < <	ABUW_2061
ABUWs038	AS (*acdP*)	*acdP*	< > <	*pqqE*
ABUWs039	IG	ABUW_2145	< < <	*rnd*
ABUWs040	AS^{P} (ABUW_2275)	ABUW_2275	> < >	ABUW_2276

ABUWs041	IG	ABUW_2313	> < >	ABUW_2314
ABUWs042	AS^{P} (ABUWs043)	ABUWs043	< > >	ABUW_2444
ABUWs043	AS^{P} (ABUWs042)	ABUW_2443	< < >	ABUWs042
ABUWs044	IG	ABUW_2520	< < <	ABUW_2521
ABUWs045	IG	ABUW_2551	< > <	ABUW_2553
ABUWs046	IG	ABUW_2597	< < >	ABUW_2598
ABUWs047	IG	ABUW_2629	< < <	ABUW_2630
ABUWs048	IG	ABUW_2699	> < <	ABUW_2700
ABUWs049	IG	ABUW_2702	> < >	ABUW_2703
ABUWs050	OL^{P} (ABUW_2704)	ABUW_2703	> < <	ABUW_2704
ABUWs051	IG	ABUW_2704	< < <	ABUW_2705
ABUWs052	IG	ABUW_2818	< < >	ABUW_2819
ABUWs053	OL^{P} (ABUW_0859)	ABUW_2859	> > <	ABUW_2860
ABUWs054	IG	*lon*	> > >	ABUW_2866
ABUWs055	OL (ABUW_2969)	*betT*	< < <	ABUW_2969
ABUWs056	IG	ABUW_2991	> > >	ABUW_2992
ABUWs057	AS (ABUW_3008)	ABUW_3008	< > <	ABUW_3009
ABUWs058	OL (ABUW_3011)	ABUW_3011	< < <	ABUW_3012
ABUWs059	IG	ABUW_3017	< < <	*ggt*
ABUWs060	IG	ABUW_3025	< > >	ABUW_3026
ABUWs061	IG	*sbcC*	> < <	ABUW_3030
ABUWs062	IG	ABUW_3080	< < <	ABUW_3081
ABUWs063	OL (*ispE*)	*ispE*	> > >	ABUW_3093
ABUWs064	IG	ABUW_3102	< > >	*ribB2*
ABUWs065	IG	*nrdB*	> < >	ABUW_3186
ABUWs066	IG	ABUW_3194	< < >	ABUW_3195
ABUWs067	IG	ABUW_3195	> < <	ABUWs068
ABUWs068	IG	ABUWs067	< < <	ABUWs069
ABUWs069	IG	ABUWs068	< < <	ABUW_3196
ABUWs070	IG*	*rho*	> > >	ABUW_3278
ABUWs071	IG	ABUW_3335	< < >	ABUW_3336
ABUWs072	AS^{P} (ABUWs073)	ABUW_3353	< > <	ABUWs073
ABUWs073	AS^{P} (ABUWs072)	ABUWs072	> < <	ABUW_3354
ABUWs074	IG	*dctA*	< < <	ABUW_3450
ABUWs075	AS^{P} (ABUW_3477)	ABUW_3477	< > >	*rne*
ABUWs076	IG	ABUW_3520	> < <	ABUWs077
ABUWs077	IG	ABUWs076	< < <	*recD*
ABUWs078	IG	ABUW_3563	> > <	*alkB*
ABUWs079	IG	ABUW_3618	> > <	*phoU*
ABUWs080	IG	ABUW_3623	> > >	*thiC*
ABUWs081	IG	ABUW_3707	> > >	ABUW_3708
ABUWs082	IG	ABUW_3724	> < <	ABUW_3725
ABUWs083	IG	ABUW_3804	< > <	ABUW_3805
ABUWs084	IG	ABUW_3890	> > >	ABUW_3891

IG: sRNA gene is located in an intergenic region; **AS:** sRNA gene is annotated antisense to existing annotation; **OL:** sRNA gene overlaps with an existing annotation in the same direction; **[P]:** sRNA and existing gene show only partial overlap (AS or OL), ***:** Transcript is located immediately upstream (or slightly overlaps) with existing transcript and might represent a riboswitch

Table S2. Possible Open Reading Frames (≥30aa) of the novel sRNA Annotation

AB5075 designation	Possible ORF #1	Possible ORF #2	Possible ORF #3
ABUWs010	MVRRRNLYYFDKRRKKSVYGPFFCVKFSFAAFWQTG*	LLLFGRLDRAAIWLAVSFVTVFLPLFSLSPLP*	
ABUWs019	MCKLLVSSRSGRTGEGHLRVCWLYLSPVCQPSFVLPP*		
ABUWs029	VCRELVRPLFLYGRAEGGHLRVCWFLCFPVC*		
ABUWs030	LFCNGSPAGGFAIVLCEPRQDRKVATVADL*		
ABUWs033	LRYFVLKLHVGIRQYHGSQNSIHHSLVGLTMLRKSDKSF*		
ABUWs037	VSMLHNIRQFFCMHIWEYDFDISHDEVRECRKCGKIHKMKSSHKMT*		
ABUWs038	MSLEYSASCFAKSSCAKQMCSICCCVKSDASWSNSLLG*	MLQHLPYAIQWLTFAVLGSAGYPEQLCKFECR*	
ABUWs043	MVAKRKRVSRLIPKKPAHPRVSHSRSSIKGTRGVTPKNVFLVRLGRRSAKTD*		
ABUWs044	MAFCVLDCKRVGHYSLPAGHIASRSSRTDFFDHIRIISSEE*		
ABUWs049	MENIEQYAHGAEHEVHVHCKRSLSTLILSLFVLIATGR*		
ABUWs052	MSFCVLDYKRVEYYSSHAGHIASRLSQTGKTKSICKILVLCVGNSFAPFFSTVGRKGDTSGVLVPLFPVCEPPFVLPP*	VCLTTRGWNTILRMLATLPAGCRRPGKQSRYAKFLFCVSGTRSPPFSLR*	MQNSCFVCRELVRPLFLYGRAEGGHLWCAGSFVSSLRTPFRSATII*
ABUWs053	MIANEVRSVILKLSLGCSPAGLIPLSRYHLFRMVFCIFRAYAYPE*		
ABUWs055#	LTPVGSGTLMIANLSSKGGTSDSDSPIWLRIFWSVVITIVSIGLLLAGSFNSMQSAVVLCGLPFSVIILLYMFGLAKALKQDDFDPNVAKKQLAISKDISIPSNCDQNKVTEVL*		
ABUWs057	MGLYVLQVLQLLNDLHHCPLPPVGFVVFAG*		
ABUWs059	LHLNSRKRRNLRSSCLRAACPLPRILVVWEPD*		
ABUWs066	MLWQNSQNQELAMHIHHFLALSAKSYNDTT*		
ABUWs071	MIDTLNIAHYPLLAGLHCQPALTGRIVKCIENLFFDVGTPVPLFCCGRTEGGRLVRAGSLLSVCQPTFVLPP*	LCVLVLYYQSVNPLSFCHHNSKLMLWQNSQYKGVGYAYSFYTSRLVLIGFDELLVYQALAFFV*	
ABUWs073	VYNRPSLVAVGRVRDTFGCADSLLSVCEPLLVLPP*	LPAGCRRPISKGIRLFPLRSSLVCTIVPRSLR*	
ABUWs075	MSIPYQTSNKEVTSRPMTVKVINQCTAHPPVKQKGIPI*	MHRSPASEAKRNSDLKVEAVLPTSQTRIEAKAKCSR*	

#: Gene is overlapping with previously annotated protein-coding ORF

*: Stop Codon

Table S3: Expression values for newly annotated sRNAs.

AB5075 designation	Expression value	AB5075 designation	Expression value
ABUWs001	12,158.95	ABUWs045	90.78
ABUWs002	877.04	ABUWs046	1,383.10
ABUWs003	482.22	ABUWs047	1,175.83
ABUWs004	102.46	ABUWs048	31.4
ABUWs005	991.3	ABUWs049	6,143.89
ABUWs006	251.92	ABUWs050	736.1
ABUWs007	302.2	ABUWs051	565.4
ABUWs008	179.71	ABUWs052	1,368.06
ABUWs009	36.13	ABUWs053	284,098.13
ABUWs010	22,418.36	ABUWs054	526.85
ABUWs011	374.51	ABUWs055	107.72
ABUWs012	22.5	ABUWs056	3,138.08
ABUWs013	142.08	ABUWs057	8.29
ABUWs014	130.92	ABUWs058	7,443.86
ABUWs015	123.34	ABUWs059	156,653.40
ABUWs016	281.19	ABUWs060	3,577.83
ABUWs017	273.43	ABUWs061	1,259.13
ABUWs018	1,100.91	ABUWs062	57,927.94
ABUWs019	1,230.10	ABUWs063	245.75
ABUWs020	79.16	ABUWs064	71.08
ABUWs021	78.93	ABUWs065	104.87
ABUWs022	93.62	ABUWs066	382.59
ABUWs023	74,148.04	ABUWs067	1,079.45
ABUWs024	2,334.28	ABUWs068	237.64
ABUWs025	145	ABUWs069	359.29
ABUWs026	1,744.26	ABUWs070	136.74
ABUWs027	49.65	ABUWs071	1,057.05
ABUWs028	41.56	ABUWs072	31.96
ABUWs029	3,457.92	ABUWs073	1,443.66
ABUWs030	128,302.13	ABUWs074	16,548.30
ABUWs031	19.69	ABUWs075	302.59
ABUWs032	168.5	ABUWs076	8,578.37
ABUWs033	59.45	ABUWs077	9,421.04
ABUWs034	985.65	ABUWs078	297.38
ABUWs035	5,937.10	ABUWs079	796.81
ABUWs036	2,939.89	ABUWs080	398.49
ABUWs037	783.5	ABUWs081	345.3
ABUWs038	26.1	ABUWs082	3,285.49
ABUWs039	43.5	ABUWs083	992.07
ABUWs040	29.85	ABUWs084	187.48
ABUWs041	107.47		
ABUWs042	1,806.25		
ABUWs043	240.85		
ABUWs044	2,495.15		

Figure S1:

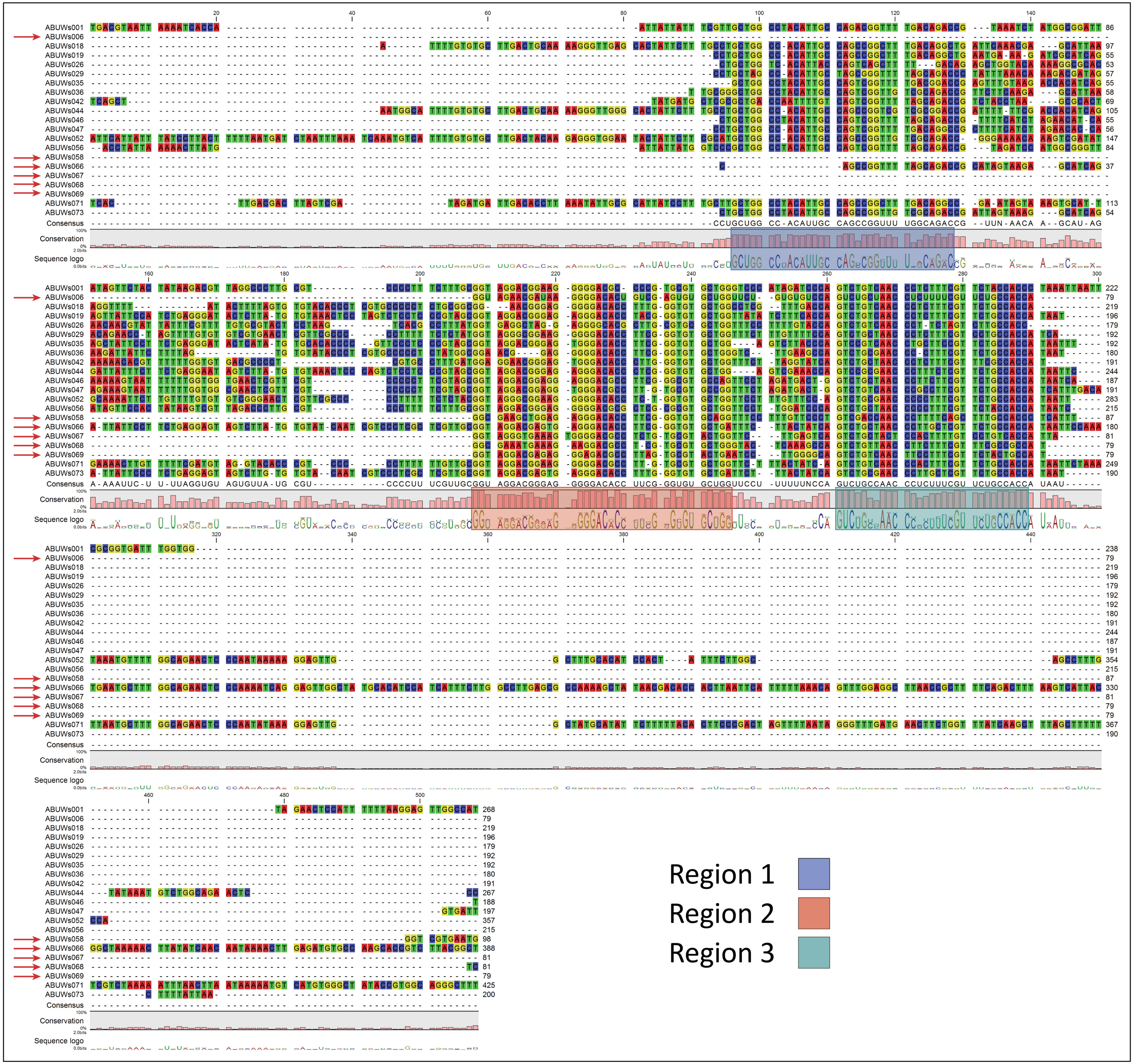

Figure S2:

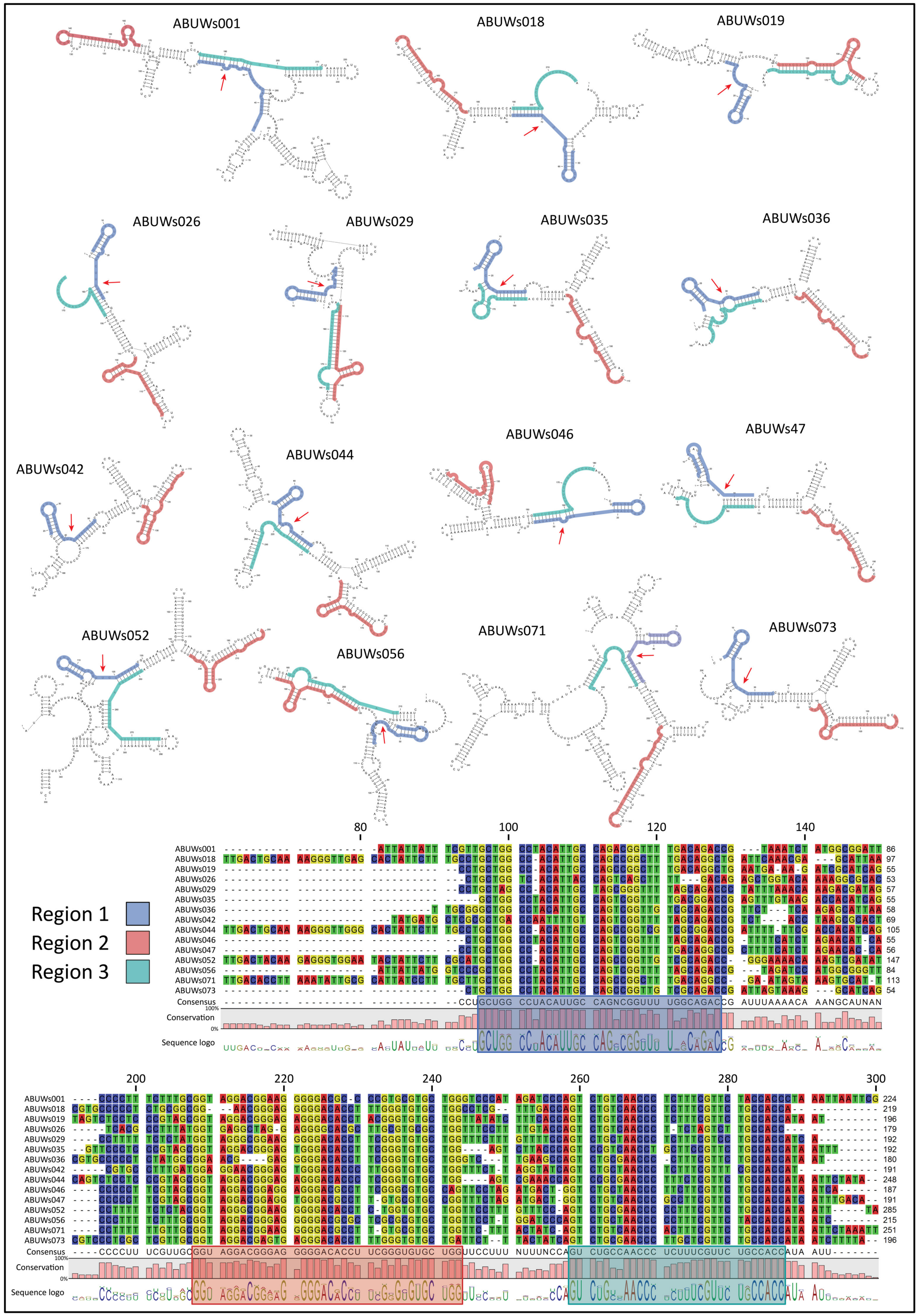

ABUWs001
ABUWs018
ABUWs019
ABUWs026
ABUWs029
ABUWs035
ABUWs036
ABUWs042
ABUWs044
ABUWs046
ABUWs47
ABUWs052
ABUWs056
ABUWs071
ABUWs073
Region 1
Region 2
Region 3
80
100
120
140
200
220
240
260
280
300
Consensus
Conservation
Sequence logo

Figure S3(a):

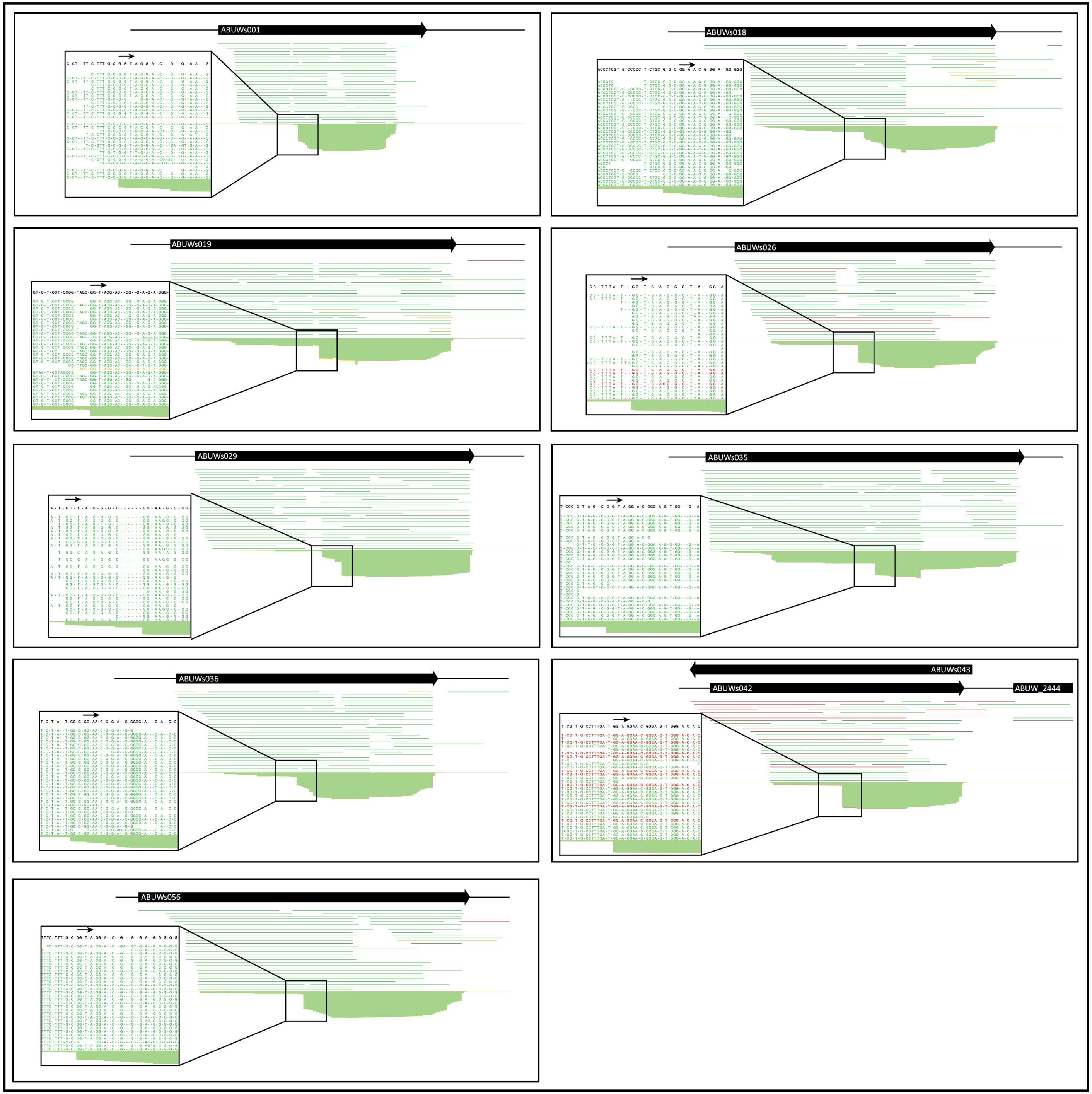
ABUWs001
ABUWs018
ABUWs019
ABUWs026
ABUWs029
ABUWs035
ABUWs036
ABUWs043
ABUWs042
ABUW_2444
ABUWs056

Figure S3(b):

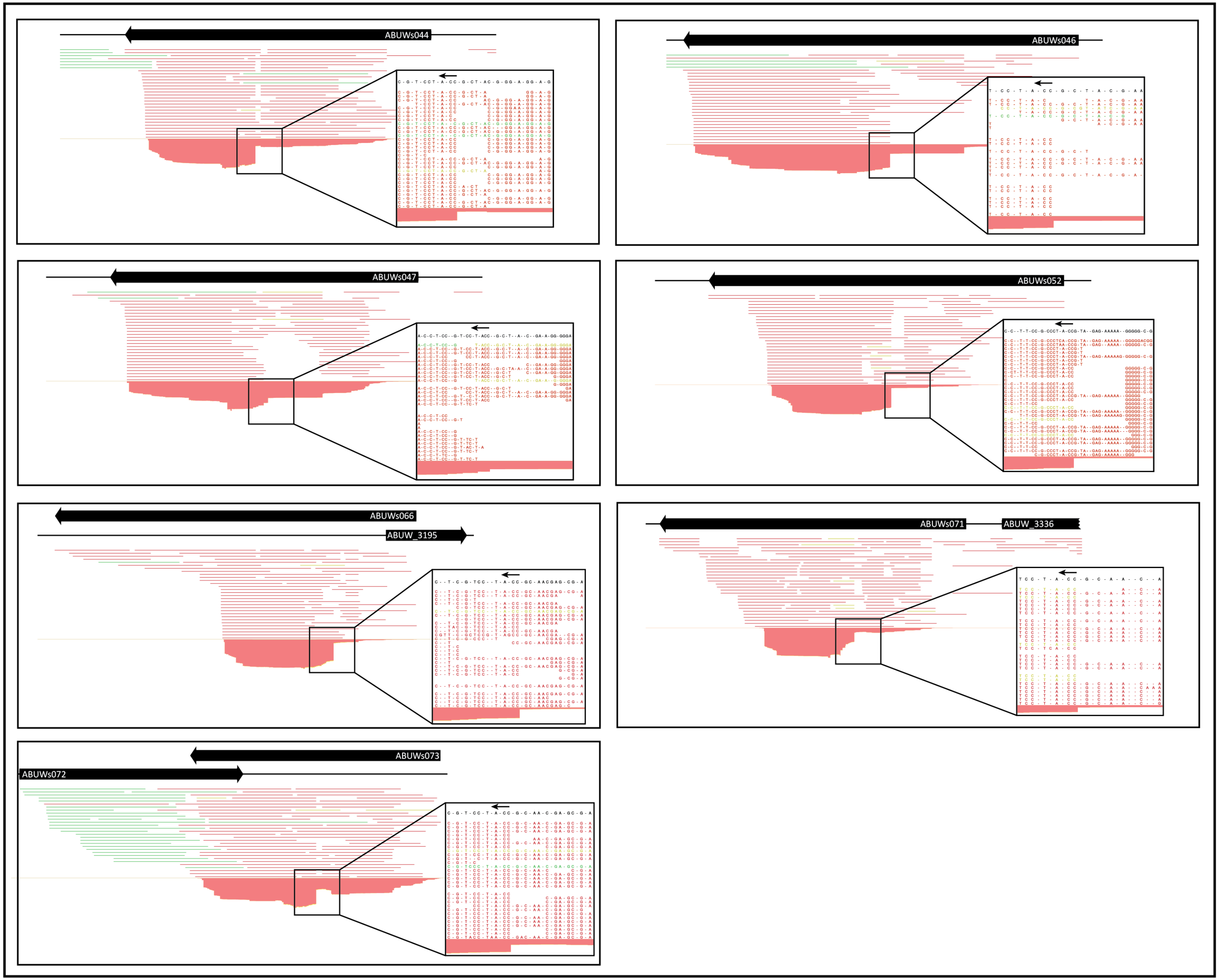
ABUWs044
ABUWs046
ABUWs047
ABUWs052
ABUWs066
ABUW_3195
ABUWs071
ABUW_3336
ABUWs073
ABUWs072

Figure S4:

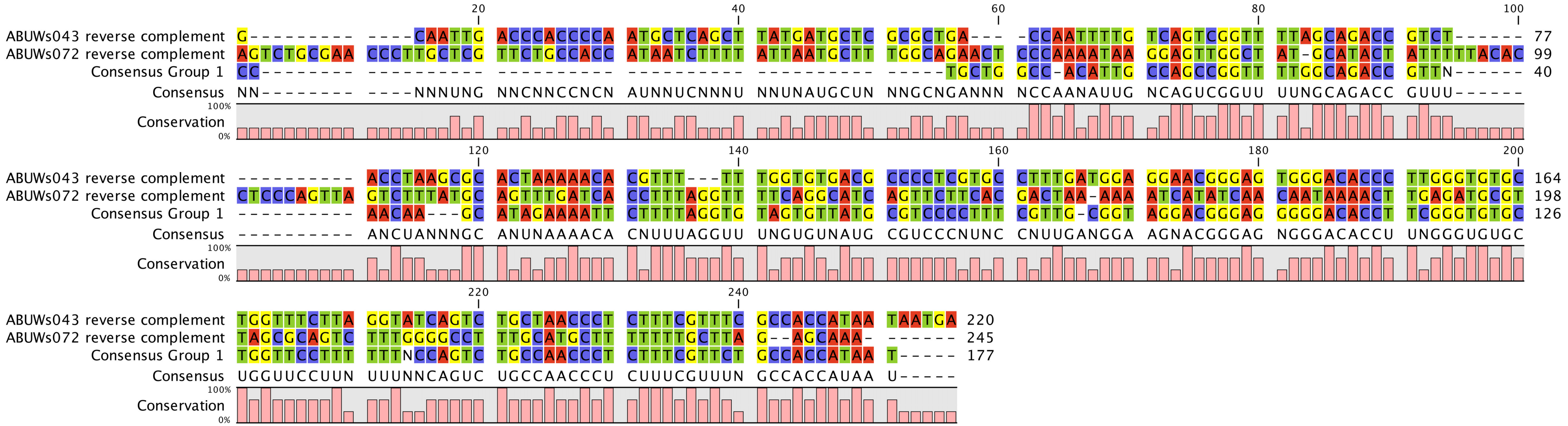

20 40 60 80 100
ABUWs043 reverse complement G--------- ----CAATTG ACCCACCCCA ATGCTCAGCT TATGATGCTC GCGCTGA--- -CCAATTTTG TCAGTCGGTT TTAGCAGACC GTCT------ 77
ABUWs072 reverse complement AGTCTGCGAA CCCTTGCTCG TTCTGCCACC ATAATCTTTT ATTAATGCTT TGGCAGAACT CCCAAAATAA GGAGTTGGCT AT-GCATACT ATTTTTACAC 99
Consensus Group 1 CC-------- ---------- ---------- ---------- ---------- -----TGCTG GCC-ACATTG CCAGCCGGTT TTGGCAGACC GTTN------ 40
Consensus NN-------- ----NNNUNG NNCNNCCNCN AUNNUCNNNU NNUNAUGCUN NNGCNGANNN NCCAANAUUG NCAGUCGGUU UUNGCAGACC GUUU------
100%
Conservation
0%
120 140 160 180 200
ABUWs043 reverse complement ---------- ACCTAAGCGC ACTAAAAACA CGTTT---TT TGGTGTGACG CCCCTCGTGC CTTTGATGGA GGAACGGGAG TGGGACACCC TTGGGTGTGC 164
ABUWs072 reverse complement CTCCCAGTTA GTCTTTATGC AGTTTGATCA CCTTTAGGTT TTCAGGCATC AGTTCTTCAC GACTAA-AAA ATCATATCAA CAATAAAACT TGAGATGCGT 198
Consensus Group 1 ---------- AACAA---GC ATAGAAAATT CTTTTAGGTG TAGTGTTATG CGTCCCCTTT CGTTG-CGGT AGGACGGGAG GGGGACACCT TCGGGTGTGC 126
Consensus ---------- ANCUANNNGC ANUNAAAACA CNUUUAGGUU UNGUGUNAUG CGUCCCNUNC CNUUGANGGA AGNACGGGAG NGGGACACCU UNGGGUGUGC
100%
Conservation
0%
220 240
ABUWs043 reverse complement TGGTTTCTTA GGTATCAGTC TGCTAACCCT CTTTCGTTTC GCCACCATAA TAATGA 220
ABUWs072 reverse complement TAGCGCAGTC TTTGGGGCCT TTGCATGCTT TTTTTGCTTA G--AGCAAA- ------ 245
Consensus Group 1 TGGTTCCTTT TTTNCCAGTC TGCCAACCCT CTTTCGTTCT GCCACCATAA T----- 177
Consensus UGGUUCCUUN UUUNCCAGUC UGCCAACCCU CUUUCGUUUN GCCACCAUAA U-----
100%
Conservation
0%

Figure S5:

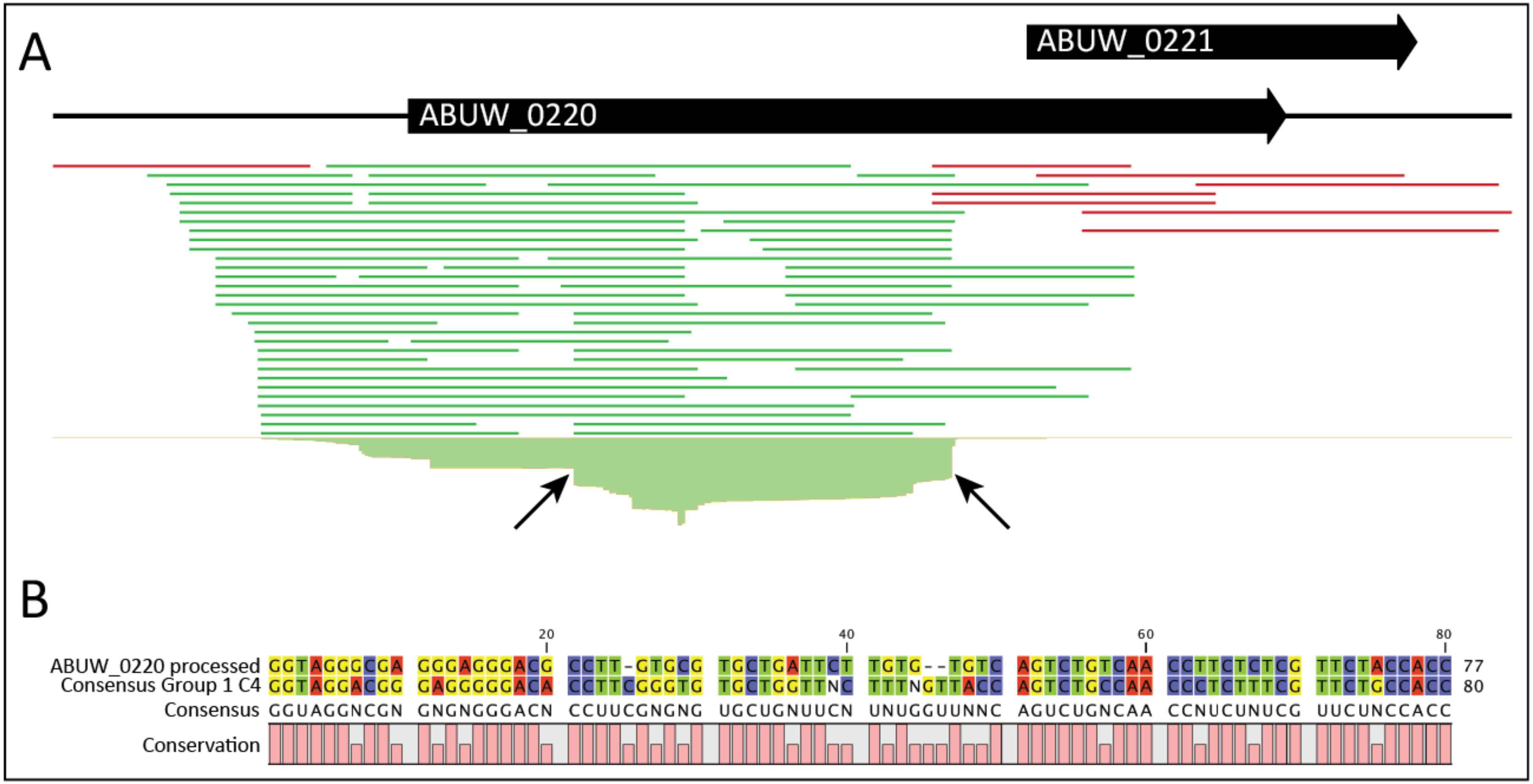
A
ABUW_0221
ABUW_0220
B
20
40
60
80
ABUW_0220 processed GGTAGGGCGA GGGAGGGACG CCTT-GTGCG TGCTGATTCT TGTG--TGTC AGTCTGTCAA CCTTCTCTCG TTCTACCACC 77
Consensus Group 1 C4 GGTAGGACGG GAGGGGGACA CCTTCGGGTG TGCTGGTTNC TTTNGTTACC AGTCTGCCAA CCCTCTTTCG TTCTGCCACC 80
Consensus GGUAGGNCGN GNGNGGGACN CCUUCGNGNG UGCUGNUUCN UNUGGUUNNC AGUCUGNCAA CCNUCUNUCG UUCUNCCACC
Conservation
